# PK-PD integration of enrofloxacin and cefquinome alone and in combination against *Klebsiella pneumoniae* using an *in vitro* dynamic model

**DOI:** 10.3389/fphar.2023.1226936

**Published:** 2023-10-06

**Authors:** Yanzhe Wei, Xuan Ji, Fuhui Zhang, Suiling Zhang, Qin Deng, Huanzhong Ding

**Affiliations:** Guangdong Key Laboratory for Veterinary Drug Development and Safety Evaluation, College of Veterinary Medicine, South China Agricultural University, Guangzhou, China

**Keywords:** enrofloxacin, cefquinome, combination therapy, PK/PD model, *Klebsiella pneumoniae*

## Abstract

**Introduction:** *Klebsiella pneumoniae* is classified as a critical pathogen in both animals and humans and infections can be fatal in chickens resulting in substantial economic losses. However, the misuse of antibiotics can also lead to drug resistance and a potential transmission chain between animals and humans. Three *K. pneumoniae* strains with different susceptibility phenotypes were chosen to study the pharmacokinetic/pharmacodynamic (PK/PD) integration of enrofloxacin (ENR) and cefquinome (CEQ) alone and in combination.

**Results:** Checkerboard assay results indicated that the combination treatment for type strain ATCC 700603 was synergistic effect with a fractional inhibitory concentration index (FICI) of ≤0.5. The other two clinical strains demonstrated an additive effect (FICI >0.5 to ≤1). Furthermore, static time-kill curves indicated that enrofloxacin and cefquinome added singly were effective in killing *K. pneumoniae* at concentrations of >2 MIC and ≥1 MIC, respectively. Additionally, the combination of enrofloxacin and cefquinome led to an enhanced antibacterial activity of cefquinome. The dynamic time-kill curves indicated that enrofloxacin and cefquinome had bactericidal and bacteriostatic activities, respectively at ≥1.5 mg/L (single-dose) and 4 mg/L (8 h split-dose) causing a decrease in bacterial counts of ≥4.45 and >2 log_10_ CFU/mL. Enrofloxacin possessed no bacteriostatic effects against *K. pneumoniae* at a constant concentration of 1× MIC. Cefquinome used in combination with 1× MIC enrofloxacin exhibited bactericidal activity at ≥4 mg/L (12 h split-dose) with reductions of ≥3.65 log_10_ CFU/mL. The PK/PD parameters were also analyzed to determine the concentration and duration of the drugs needed to reduce bacteria by 3 log_10_ CFU/mL. For enrofloxacin alone, the AUC_24_h/MIC was 23.29 h and the C_max_/MIC was 3.18. For cefquinome alone, the %T > MIC was 48.66 and when used in combination with enrofloxacin was 18.04. The combined use of cefquinome and enrofloxacin can increase the antibacterial activity of cefquinome against *K. pneumoniae* under a 12-h split-dose regimen regardless of individual drug susceptibility.

**Discussion:** The static and dynamic time-kill curves indicated that enrofloxacin exhibited concentration-dependent activity, while cefquinome exhibited time-dependent activity. In the *in vitro* dynamic model, enrofloxacin alone exhibited better antimicrobial effects against *K. pneumoniae* compared to cefquinome alone. However, the antibacterial effect of cefquinome can be enhanced by combining it with enrofloxacin. These findings suggest a potentially effective approach for combating *K. pneumoniae* infections.

## Introduction

*Klebsiella pneumoniae* is a Gram-negative bacterium that is present in numerous ecological niches in humans and animals including the respiratory and digestive tracts and even hair. It also has a wide distribution in natural environments such as soil and water. This bacterium is a common zoonotic pathogen and infections can result in pneumonia, meningitis, sepsis, endocarditis and wound infection (Podschun and Ullmann, 1998; De Rosa et al., 2015). *K*. *pneumoniae* isolates commonly possess numerous plasmid-encoded antimicrobial resistance genes and this suggests that its wide ecological distribution and diverse DNA composition are critical factors in its ability to transfer antibiotic resistance genes (ARGs) from environmental microorganisms to clinically important pathogens (Wyres and Holt, 2018).

There is a widespread presence of antibiotic-resistant *K*. *pneumoniae* on poultry and livestock farms (Wu et al., 2016; Li et al., 2022; Yudhanto et al., 2022). For instance, a one-year cow mastitis investigation conducted on a large farm in Japan found that the most common bacterial isolates from 1,549 samples (952 cows) were non-aureus staphylococci (27.6%), *Escherichia coli* (18.9%) and *K*. *pneumoniae* (12.3%) (Taniguchi et al., 2021). Another study of hospitalized patients and farmed pigs, chickens, cows and sheep in Henan Province, China identified 189 non-repetitive *K*. *pneumoniae* strains and the prevalence of multiple drug resistance was 93.6% for pigs, 90.4% for humans, 88.9% for chickens, 52.0% for cows and 50.0% for sheep (Yang et al., 2019). Increased resistance of *K*. *pneumoniae* to commonly used antibiotics can lead to decreased treatment effectiveness. A combination of cephalosporins and quinolones have been found to exert synergistic or partially synergistic effects against these infections (Mackay et al., 2000).

Enrofloxacin is a fluoroquinolone that is commonly used in veterinary clinical treatment due to its good pharmacokinetic (PK) properties and excellent activity against Gram-negative aerobic bacteria and some Gram-positive bacteria (Pallo-Zimmerman et al., 2010). Enrofloxacin inhibits bacterial DNA gyrase and topoisomerase and this interferes with the distribution of replicated DNA to daughter cells and results in a bactericidal effect. Enrofloxacin has a longer half-life in pigs (9.3 h) and chickens (6.9 h) than in rats (1.6 h) (Bimazubute et al., 2010; Yang et al., 2015; Atef et al., 2020). Cefquinome is a fourth-generation cephalosporin used exclusively for animals. It has a strong antibacterial activity against both Gram-positive and Gram-negative bacteria and a good clinical therapeutic effect on animal respiratory diseases (Brown et al., 1999). Cefquinome kills bacteria by interfering with cell wall synthesis and altering cell membrane permeability. Its half-life is longer in pigs (4.92 h) than in chickens (1.35 h), ducks (1.79 h) and rabbits (1.04 h) (Xie et al., 2013).

The *in vitro* PK/PD integration model is often used to study dynamics between drugs and bacterial growth using *in vitro* steady-state cultures. This can guide the design of dosage regimens by calculating and simulating the antibacterial effects and PK/PD parameters (Liu et al., 2005; Vaddady et al., 2019). *In vitro* PK/PD models are relatively simple and more economical than *in vivo* experiments. They are also easy to operate and can directly describe the dynamic interaction between drugs and bacteria (Zhang H et al., 2022). However, PK/PD interactions of animal-specific antibiotics against *K*. *pneumoniae* have not been reported.

The purpose of this study was to first determine the minimum inhibitory concentration (MIC) and the fractional inhibitory concentration index (FICI) of enrofloxacin and cefquinome against *K*. *pneumoniae*. Secondly, we wanted to establish static time–kill curves in defined artificial culture media and quantitatively analyze and fit the relationship between bacterial growth and drug concentration through the E_max_ model. We then studied relationships between the PK/PD index and the effects of enrofloxacin and cefquinome alone or in combination using an *in vitro* dynamic PK/PD model. Finally, we examined the emergence of enrofloxacin-resistant bacteria during the dynamic *in vitro* process and screened for the presence of specific mutations. This study can be used to set dosing guidelines for the treatment of *K*. *pneumoniae* infections in animals.

## Materials and methods

### Materials

The Laboratory of Veterinary Pharmacology within South China Agricultural University (Guangzhou, China) provided all four bacterial strains for this study. *E*. *coli* ATCC 25922 and *K*. *pneumoniae* ATCC 700603 were used as quality control standards for drug susceptibility testing. *K*. *pneumoniae* clinical isolates ZJ42 and CLS2 were confirmed as *K*. *pneumoniae* using 16S rDNA sequencing with the 3730XL system (Thermo Fisher, Pittsburg. PA, United States). Sequence data was compared and identified using the NCBI database (https://www.ncbi.nlm.nih.gov/). Enrofloxacin (≥98%) and cefquinome (≥97%) were purchased from Yuanye Bio-Technology (Shanghai, China). MHII broth and MacConkey agar were purchased from Hopebio Biotechnology (Qingdao, China).

### MIC determination and checkerboard assay

The MICs for enrofloxacin and cefquinome for *K*. *pneumoniae* strains was determined using a broth-microdilution method using supplemented MHII broth as previously described (Kristoffersson et al., 2020). In brief, enrofloxacin and cefquinome were diluted in a two-fold series in MHII in Costar 3599 96-well plates (Corning, Corning, NY, United States) at 100 μL per well. An equal volume of an exponential-phase culture of *K*. *pneumoniae* diluted with medium was added to achieve a cell density of 10^5^ colony-forming units (CFU)/mL. *E*. *coli* ATCC 25922 strain was used as the quality control. MIC measurements for each drug were repeated thrice. MIC values for these isolates were determined following the CLSI guidelines (Clsi, 2018).

The interactions between enrofloxacin and cefquinome were evaluated using the checkerboard assay method with each antibiotic in a range of 4 × MIC to 1/16 × MIC using 5 × 10^5^ CFU/mL. Turbidity was checked after 16–18 h of incubation at 37°C. The FICI was calculated using the following formula:

$$\begin{array}{c} FICI=\frac{\left(MIC\;of\;\text{enrofloxacin }\;in\;combination\;with\;\text{cefquinome}\right)}{\left(MIC\;of\;\text{enrofloxacin}\;alone\right)}\\ +\frac{\left(MIC\;of\;\text{cefquinome }\;in\;combination\;with\;\text{enrofloxacin}\right)}{\left(MIC\;of\;\text{cefquinome}\;alone\right)} \end{array}$$

The FICI was interpreted as follows: FICI ≤0.5 denotes synergy; FICI >0.5 to ≤1 denotes additivity; FICI >1 to ≤4 denotes no interaction; FICI >4 denotes antagonism. For each strain, the checkerboard assay was repeated thrice (Odds, 2003).

### Exposure to a static concentration of antibiotic

Static time–kill data were generated following CLSI recommendations (Clsi, 1999). *K*. *pneumoniae* cultures (5 mL) containing 10^6^ CFU/mL were exposed to enrofloxacin (0, 0.5, 1, 2, 4 and 8 × MIC), cefquinome (0,0.5,1,2,4 and 8 × MIC) and cefquinome 0, 0.5, 1, 2, 4 and 8 × MIC with enrofloxacin at 0.5 × MIC. Cultures were incubated at 37°C with shaking. Aliquots were taken from each bottle at 0,1,3,6,9,12 and 24 h for CFU determinations. In brief, 20 μL aliquots of 10-fold dilutions were spread-plated on agar and incubated for 24 h at 37°C. The limit of detection was 50 CFU/mL. Bactericidal and bacteriostatic activities were defined as >3-log_10_ and >2-log_10_ reduction in CFU/mL, respectively (Li et al., 2016; Zhang L et al., 2022).

The E_max_ model is represented as:

$$E=\frac{Emax\times C_{e}^{N}}{{EC}_{50}^{N}+C_{e}^{N}}$$

where *E* is the kill rate, *E*_*max*_ is the maximum kill rate in a certain period of time, *C*_*e*_ is the drug concentration, *N* is the Hill coefficient that describes the steepness of the kill rate-effect curve, *EC*_*50*_ is the drug concentration that produced 50% of the maximum kill rate. The correlation coefficient *R* ^*2*^ represents the degree of similarity between the experimental (observed) value and the *E*_*max*_ (predicted) value (Huang et al., 2020).

### *In vitro* dynamic PK/PD model

An *in vitro* one-compartment PK/PD model of infection was employed to examine the antimicrobial efficacy of enrofloxacin and cefquinome alone and in combination against *K*. *pneumoniae* over 24 h (Xiao et al., 2015; Huang et al., 2020). The apparatus setup included a storage compartment for fresh MHII broth, a central compartment for drug-bacteria interaction and a waste storage compartment. The central compartment contained 200 mL sterile culture medium. The flow rates of the peristaltic pump were set to 0.35 mL/min (enrofloxacin, t_1/2β_ = 6.53 h) and 1.49 mL/min (cefquinome, t_1/2β_ = 1.55 h) to simulate the drug PK in chicken plasma. Based on the maximum concentration (C_max_) of the drugs in chicken plasma, different dosages of enrofloxacin and cefquinome were designed for each strain for dynamic modeling: The enrofloxacin monotherapy groups contained single doses of 0.2, 0.75, 1.25, 1.5, and 2 mg/L and split doses of 1, 1.5, and 2 mg/L given twice a day. The cefquinome monotherapy groups were given split doses of 2 and 4 mg/L twice a day and split doses of 1.5, 2, 3, and 4 mg/L given three times a day. A combination group with a fixed concentration of enrofloxacin at 1 × MIC and a single dose of 4 mg/L cefquinome given twice a day and split doses of 0.75, 1.25, 2, and 4 mg/L given twice a day. For combination therapy, enrofloxacin was administered by continuous infusion to simulate a long t_1/2β_ in the model. A series of studies using different doses of cefquinome and 1 × MIC enrofloxacin were conducted to evaluate the PK/PD target of *K*. *pneumoniae* in the presence of enrofloxacin. Each experiment included bacterial control groups treated with enrofloxacin or cefquinome only and the quality control bacteria. We withdrew 0.1 mL samples prior to antibiotic addition (0 h) as well as at 1, 3, 6, 9, 12, 15 and 24 h after administration for CFU counting and 1 mL for determination of the drug levels in the growth medium. PK samples were stored at −20°C until analysis.

### Quantification of enrofloxacin and cefquinome in the medium

Enrofloxacin levels in culture media were measured by high performance liquid chromatography (HPLC) (Agilent Technologies. Santa Clara, CA, United States). Separation was achieved on a Phenomenex C18 column (4.6 × 250 mm, 5 μm; Torrance, CA, United States). The mobile phase was 0.05 M citric acid, 0.1 M ammonium acetate: acetonitrile (86:14, v/v). The injection volume was 50 μL. A calibration curve was established with six enrofloxacin concentrations (0.001–0.2 μg/mL). PK parameters were calculated using WinNonlin 6.1 (Pharsight, Mountain View, CA, United States).

Cefquinome levels in the medium were measured by HPLC tandem mass spectrometry using the Agilent system. Separation was achieved on a Phenomenex C18 column (2.0 × 150 mm, 5 μm). Acetonitrile was used to extract the culture medium (Zhang et al., 2018b). The HPLC mobile phase consisted of solution A (0.1% formic acid) and solution B (acetonitrile) at a flow rate of 0.25 mL/min. The elution gradient was: 0–1 min, 5% B; 1–5.5 min, 60% B; 5.5–10 min, 5% B. The injection volume was 5 μL. A calibration curve was established with six cefquinome concentrations (0.001–0.2 μg/mL). PK parameters were calculated using WinNonlin 6.1.

### Fitting and analyses of dynamic time–kill curves

The results of dynamic modeling studies were analyzed using the inhibitory sigmoidal maximum effect (E_max_) PD model. The PK/PD parameters of the area under the concentration—time curve over 24 h (AUC_0–24h_), C_max_ and the cumulative percentage of time over a 24 h period in which the concentration exceeded the MIC (%T > MIC) were calculated according to a one-compartmental analysis using WinNonlin. T-tests were conducted for data using SPSS software (IBM, Armonk, NY, United States). *p* < 0.05 was considered statistically significant.

The inhibitory sigmoidal E_max_ model was:

$$E=E_{\max}-\frac{\left(E_{\max}-E_{0}\right)\times C_{e}^{N}}{{EC}_{50}^{N}+C_{e}^{N}}$$

where E is the antibacterial effect; E_max_ is the change in the control group (log_10_ CFU/mL) from 0 h to 24 h; E_0_ is the maximum value of the antibacterial effect; Ce is the value of a PK/PD index (C_max_/MIC, AUC_24h_/MIC, and %T > MIC); EC50 is the corresponding PK/PD index that produces a 50% reduction of the maximum antibacterial effect; N is the Hill coefficient (Huang et al., 2020; Zhang H et al., 2022).

### *In vitro* competition tests between resistant and sensitive bacteria

Enrofloxacin-resistant and the original susceptible strains were mixed in a 1:1 ratio and the relative fitness value (W) based on the results of paired competitive growth were calculated as previously described (Martel et al., 2001).

$$W=\frac{\ln\left(\frac{RF}{RI}\right)}{\ln\left(\frac{SF}{SI}\right)}$$

RI and SI represent the number of resistant bacteria and sensitive bacteria in the mixed solution before each transmission and RF and SF the numbers after each transmission, respectively. The relative fitness value of the resistant strain to the original sensitive bacteria was obtained by calculation.

### PCR gene amplification

Cultures of test strains in MHII broth were incubated for 12 h and total DNA was extracted using the TIANamp Bacteria DNA Kit (Tiangen, Beijing, China). PCR amplification was performed for the quinolone resistance-determining region (QRDR) genes *gyr*A, *gyr*B, *par*C and *par*E for both resistant and initially sensitive strains and for the resistance genes *qnr*A, *qnr*S, and *aac* (6′)-Ib-cr. PCR primers were as follows: 5′-AAA​TCT​GCC​CGT​GTC​GTT​GGT-3′ and 5′-GCC​ATA​CCT​ACG​GCG​ATA​CC-3′ for *gyr*A, 5′-ATG​GAT​AAA​GAA​GGC​TAC​AGC​A-3′ and 5′-TCG​ACG​TCC​GCA​TCG​GTC​AT-3′ for *gyr*B, 5′-ATG​TAC​GTG​ATC​ATG​GAC​AG-3′ and 5′-ATT​CGG​TGT​AAC​GCA​TGG​C-3′ for *par*C, and 5′-GAC​CGA​AAG​CTA​CGT​CAA​CC-3′ and 5′-GTT​CGG​ATC​AAG​CGT​GGT​TT-3′ for *par*E (Nam et al., 2013). The other antibiotic resistance genes were: 5′-ATT​TCT​CAC​GCC​AGG​ATT​TG-3′ and 5′-GAT​CGG​CAA​AGG​TTA​GGT​CA-3′ for *qnr*A, 5′-ACG​ACA​TTC​GTC​AAC​TGC​AA-3′ and 5′-TAA​ATT​GGC​ACC​CTG​TAG​GC-3′ for *qnr*S, and 5′-TTG​CGA​TGC​TCT​ATG​AGT​GGC​TA-3′ and 5′-CTC​GAA​TGC​CTG​GCG​TGT​TT-3′ for *aac* (6′)-lb-cr (Guo et al., 2016). The PCR was performed in a 50 μL reaction volume that contained 25 μL of Premix Taq (Takara, Dalian, China), 10 μM of each primer and 1 μL of template DNA. The PCR conditions consisted of an initial denaturation at 95°C for 5 min and 35 cycles of 94°C for 50 s, 55°C for 40 s and 72°C for 1 min followed by a final extension at 72°C for 5 min in a thermocycler. The DNA sequences were annotated using BLAST (http://blast.ncbi.nlm.nih.gov) to identify the gene subtypes.

## Results

### Susceptibility testing

We initially evaluated the activity of enrofloxacin and cefquinome against *K*. *pneumoniae* using MIC and checkerboard assays. The MICs for *K*. *pneumoniae* strains ATCC 700603, CLS2 and ZJ42 were 64, 0.2 and 0.13 μg/mL, respectively. The FICI values also indicated a synergistic effect of the enrofloxacin/cefquinome combination against ATCC 700603 while an additive effect was noted for the clinical strains ZJ42 and CLS2. Enrofloxacin used at 0.5 ×MIC with cefquinome reduced the MIC 32-, 4- and 8-fold for strains ATCC 700603, CLS2 and ZJ42, respectively (Table 1).

**TABLE 1:** Minimum inhibitory concentration (MIC) and fractional inhibitory concentration index (FICI) *in vitro* susceptibility tests.

Strain	MIC - enrofloxacin (mg/L)	MIC - cefquinome (mg/L)	FICI	MIC—(cefquinome/enrofloxacin 0.5 × MIC) (mg/L)
ATCC 25922^a^	0.06	0.06	—	—
ATCC 700603^b^	64	32	0.33	1
ZJ42^b^	0.13	0.13	0.75	0.06
CLS2^b^	2	0.13	0.75	0.03
ZJ42-1.5s^c^	1	0.13	0.63	0.02

^a^ *E*. *coli*.

^b^ *K*. *pneumoniae*.

^c^ ZJ42 derivative. Total and ZJ42-1.5s strains were induced from the *in vitro* dynamic model.

### Static time–kill curves

**FIGURE 1:**
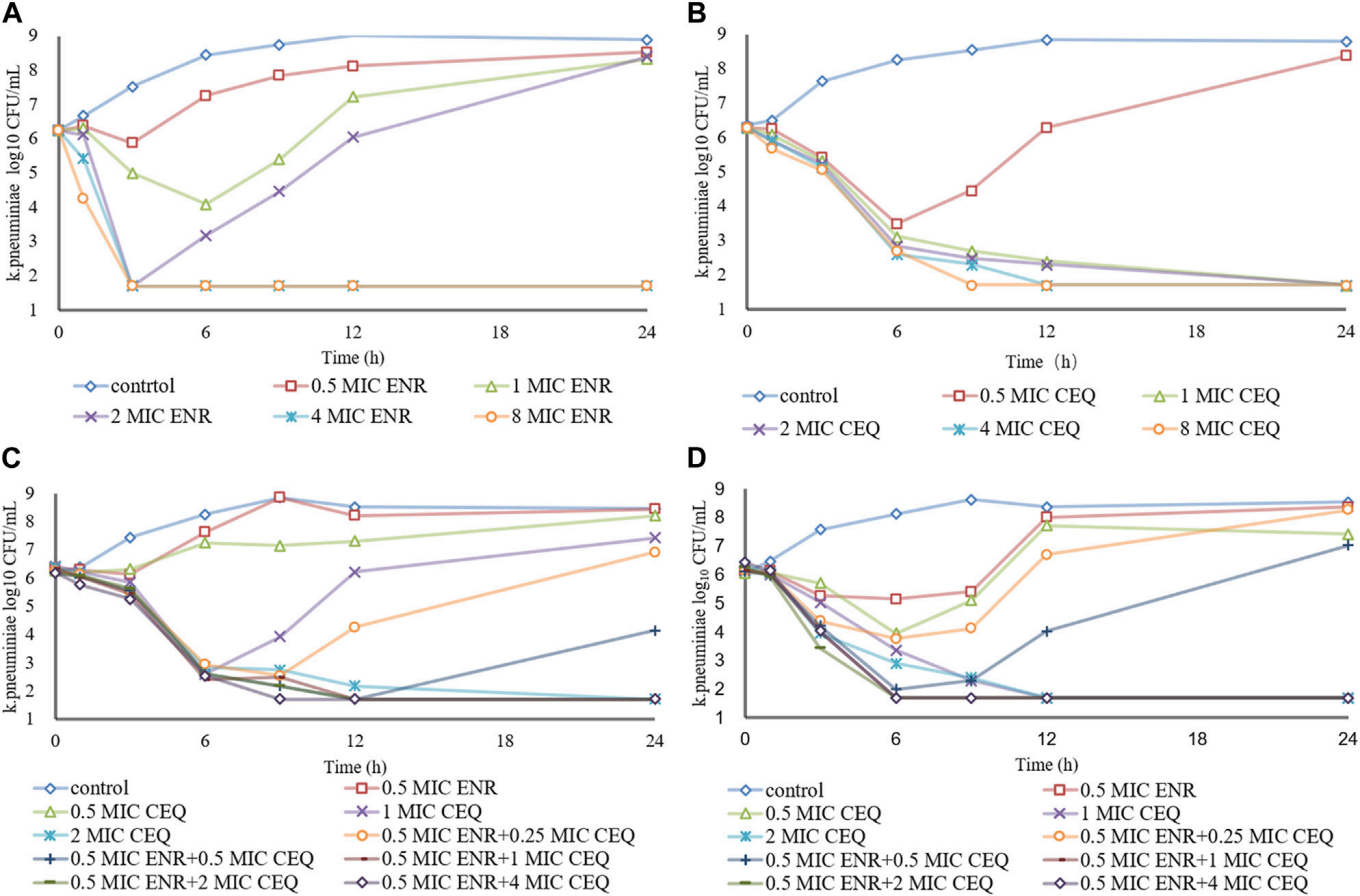
Time–kill studies. Enrofloxacin (ENR) and cefquinome (CEQ) used alone and in combination against *K*. *pneumoniae* at constant concentrations **(A)** Enrofloxacin alone with strain ZJ42 **(B)** Cefquinome used alone with strain ZJ42. Cefquinome in combination with enrofloxacin with strains **(C)** ZJ42-1.5s and **(D)** CLS2.

We also measured the variations in the numbers of *K*. *pneumoniae* bacteria cultured in the presence of differing concentrations of enrofloxacin and cefquinome. Enrofloxacin and cefquinome added at ≤ 2 × MIC and ≤1 × MIC, respectively, resulted in CFU numbers that slowly increased relative to the initial inoculum. However, at >2 × MIC and ≥1 × MIC respectively, the CFU numbers fell below the detection limits of the assay in 24 h (Figures 1A, B). We then determined the effects of cefquinome at a fixed 0.5 × MIC of enrofloxacin that possessed no bactericidal effect when used alone. In this process we identified a ZJ42 derivative (ZJ42-1.5s) that increased its MIC to enrofloxacin in the *in vitro* dynamic model that survived for 30 h (Figure 1C). In addition, CLS2 was enrofloxacin resistant and in the presence of 0.5 ×MIC enrofloxacin, an inhibitory effect was observed at cefquinome concentrations <1 MIC while no bactericidal effect was observed at <1 MIC for cefquinome alone (Figure 1D). We used SPSS software to determine the *p*-value of the static time-kill curve with respect to control. The *p*-value of enrofloxacin and cefquinome alone or in combination were between 0.003–0.033, all <0.05.

**FIGURE 2:**
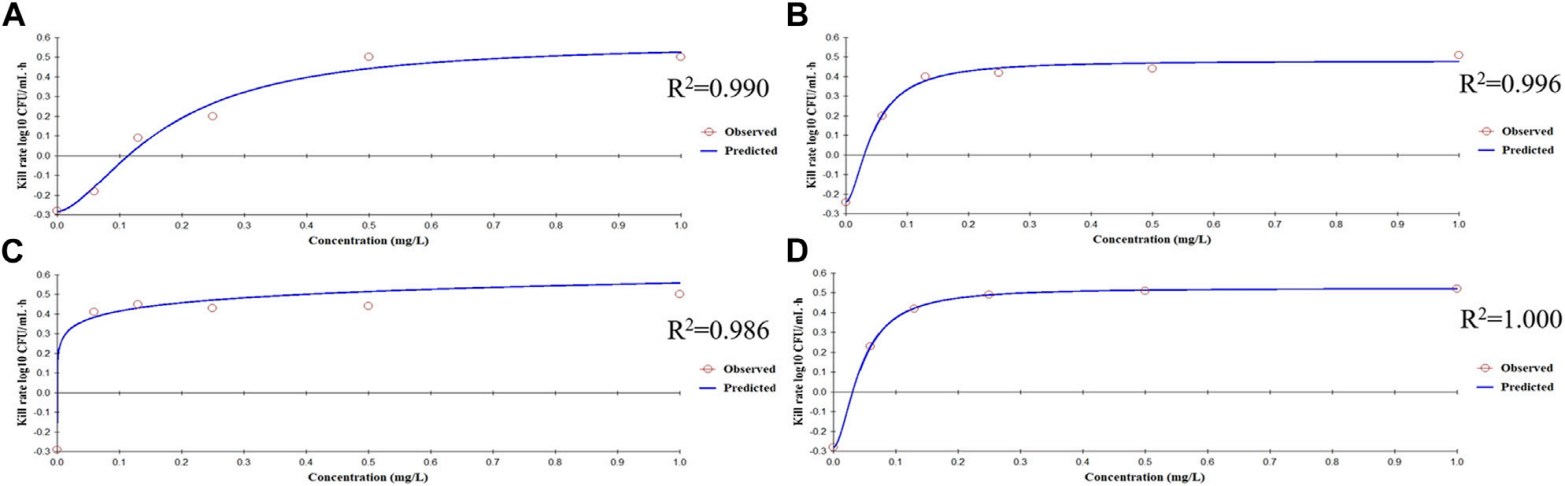
Relationships between the kill rate and concentration obtained from the E_max_ model at 0–9 h **(A)** Enrofloxacin used alone with strain ZJ42 **(B)** Cefquinome used alone with strain ZJ42. Cefquinome in combination with enrofloxacin for the strains **(C)** ZJ42-1.5s and **(D)** CLS2.

The bactericidal activities of enrofloxacin and cefquinome alone or in combination could only be calculated for 0–9 h because no CFU were detected after 9 h incubation. For this time interval, the drug concentration was positively correlated with the kill rate (Figure 2). Additionally, the E_max_ values for enrofloxacin alone and in combination were higher than that of cefquinome alone, while the EC_50_ of cefquinome alone and enrofloxacin combined with cefquinome were lower than that of enrofloxacin alone under similar Hill coefficient conditions (Table 2).

**TABLE 2:** Estimation of the kill-rate parameter derived from the E_max_ model which fitted the data from the *in vitro* static time–kill curve at an interval of 0–9 h.

Group	E_max_ (log_10_ CFU/mL/h)	EC_50_ (μg/mL)	Hill coefficient	R^2^
A	0.57	0.18	1.64	0.990
B	0.48	0.05	1.71	0.996
C	1.25	0.90	0.18	0.986
D	0.52	0.04	1.78	1.000

E_max_, maximum value of the kill rate at an interval of 0–9 h; EC_50_, concentration at which 50% of the maximum kill rate was reached; *R* ^2^, correlation coefficient of the relationship between the experimental value and predicted value (A) Enrofloxacin used alone with strain ZJ42 (B) Cefquinome used alone with strain ZJ42. Cefquinome in combination with enrofloxacin for the strains (C) ZJ42-1.5 s and (D) CLS2.

### PK/PD modeling of enrofloxacin and cefquinome alone against *K*. *pneumoniae*

**FIGURE 3:**
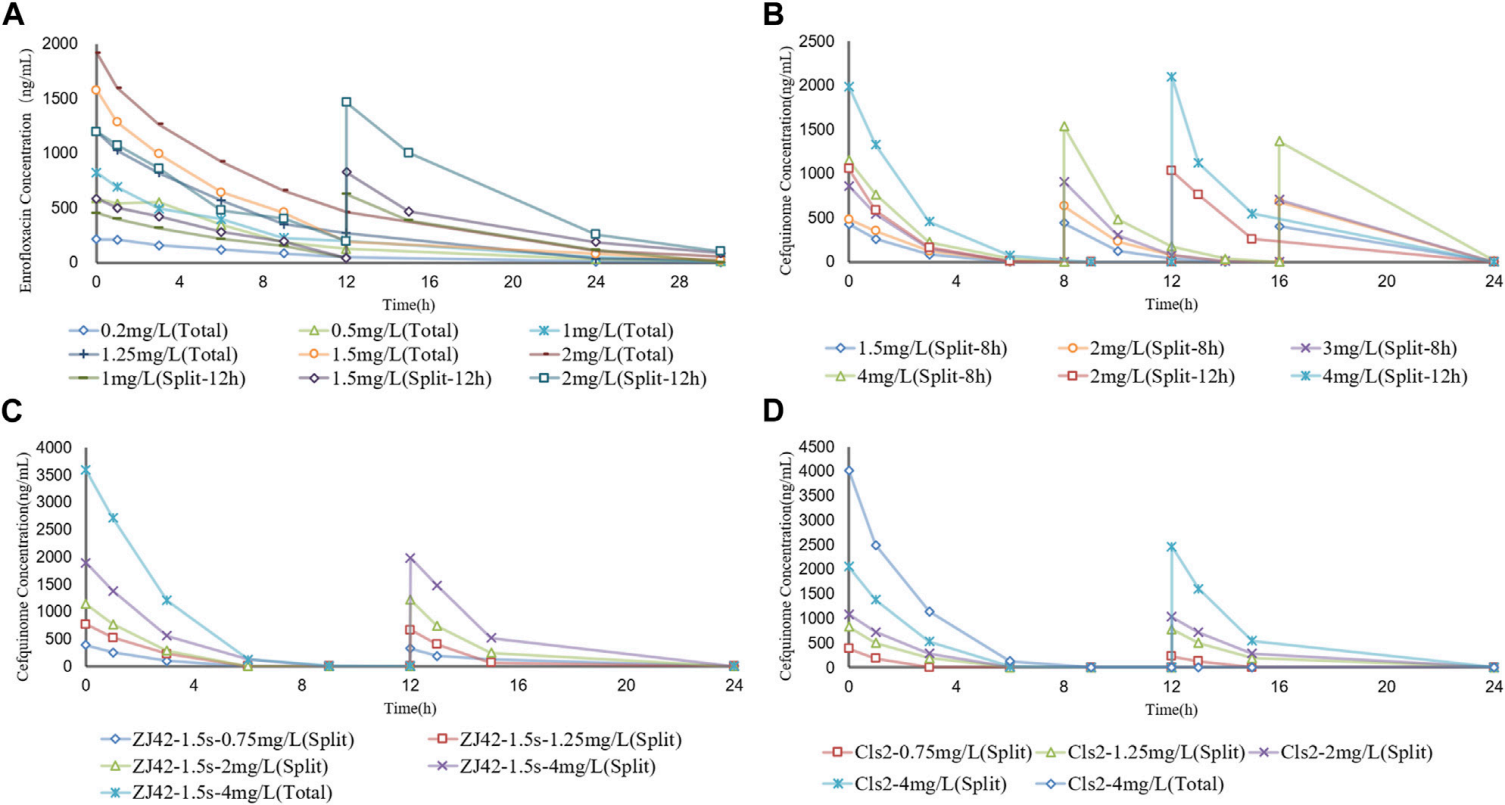
Concentration–time curves of enrofloxacin and cefquinome in the dynamic model of single drug and combination drug **(A)** Enrofloxacin used alone with strain ZJ42 **(B)** Cefquinome used alone with strain ZJ42. Cefquinome in combination with enrofloxacin for strains **(C)** ZJ42-1.5s and **(D)** CLS2. Total, single dose groups. Split, 8 h or 12 h split dosage groups.

**FIGURE 4:**
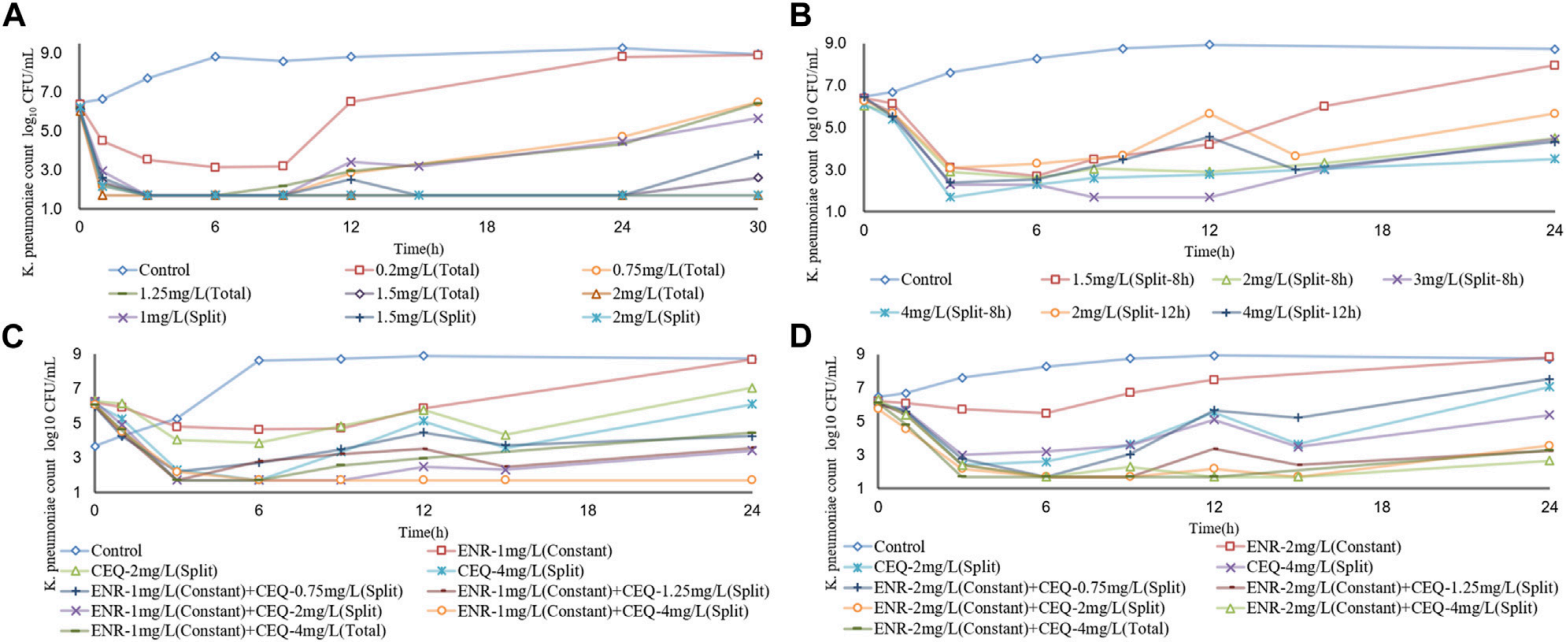
Dynamic time–kill curves of enrofloxacin and cefquinome used alone and in combination against *K*. *pneumoniae* **(A)** Enrofloxacin used alone used with strain ZJ42 **(B)** Cefquinome used alone with strain ZJ42. Cefquinome in combination with enrofloxacin for strains **(C)** ZJ42-1.5s and **(D)** CLS2. Total, single dose group. Split-8 h, 8-h split dose group. Split and Split-12h, 12-h split dose groups.

To simulate antibiotic exposure in clinical situations, we designed 8 enrofloxacin and 6 cefquinome dosage groups based on previous clinical studies (Xie et al., 2013; Xiao et al., 2018; Atef et al., 2020). Enrofloxacin levels at ≥ 1.5 mg/L resulted in CFU counts below the detection limit for 24 h. At enrofloxacin levels of 0.25–1.5 mg/L, the CFU count reductions were approximately equal. The 8 h segmented dose for cefquinome at > 1.5 mg/L exhibited inhibitory effects on the ZJ42 strain. When the 8 h and 12 h segmented dosages were 4 mg/L, the CFU count reduction was in the range of 2 - 3 log_10_ CFU/mL (Figures 3A, B and Figures 4A, B). We used SPSS software to determine the *p* values of the dynamic time-kill curve with respect to control. The *p* values of enrofloxacin and cefquinome alone or in combination were between 0.000–0.017, all <0.05.

**FIGURE 5:**
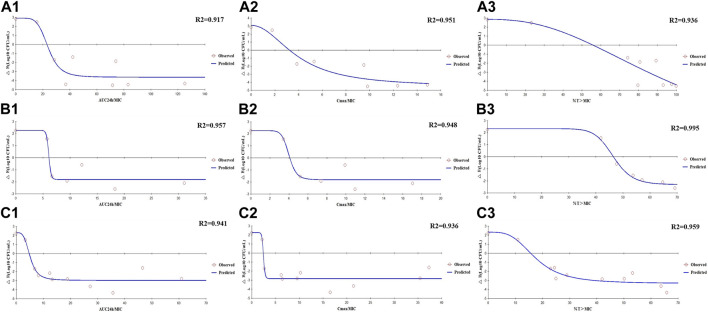
E_max_ relationships for the PK/PD parameters of enrofloxacin and cefquinome used alone and in combination *versus* antibacterial effects **(A1–A3)** Enrofloxacin used alone against strain ZJ42. **(B1–B3)** Cefquinome used alone against strain ZJ42. **(C1–C3)** Cefquinome in combination with enrofloxacin against ZJ42-1.5s and CLS2 strains.

The E_max_ relationships of the PK/PD parameters *versus* the antibacterial effect for enrofloxacin and cefquinome were then calculated. Strain ZJ42 displayed *R* ^2^ values for enrofloxacin of AUC_24h_/MIC, C_max_/MIC, and %T > MIC with an antibacterial effect was 0.917, 0.951 and 0.936, respectively. For cefquinome these values were 0.957, 0.948 and 0.995, respectively (Figure 5). Based on the fitting results of PK/PD parameters and the law of Time-kill studies, enrofloxacin used alone displayed a clear positive correlation between concentration and time while this was not the case for cefquinome alone. Enrofloxacin used alone resulted in C_max_/MIC with the highest *R* ^2^ while %T > MIC fitting to the 3 PK/PD parameters did not correlate. When cefquinome was used alone, %T > MIC generated the highest *R* ^2^ and the fitting curve of %T > MIC was the most reasonable. These data indicated that enrofloxacin has a concentration-dependent bactericidal effect on *K*. *pneumoniae* while cefquinome has a time-dependent effect (Figures 4, 5; Table 4)

In the dynamic PK/PD model of enrofloxacin against *K*. *pneumoniae*, MIC increases were > 2-fold for 3 dosage groups. The relative fitness value (W) at 1.25 mg/L and the split dosage group of 1.5 mg/L approached 1 at 24 h and 30 h. Interestingly, none of the 3 *K*. *pneumoniae* strains exhibited QRDR mutations but low-level resistance was attributed to *aac* (6’)-lb-cr (Table 3).

**TABLE 3:** MIC values of drug-resistant bacteria, the types of drug resistance and the relative fitness value (W).

Drug	Bacterial numbering	MIC (mg/L)					Resistant type	W
		Enrofloxacin	Enrofloxacin + CCCP	The multiplier of enrofloxacin MIC	Cefquinome	The multiplier of cefquinome MIC		
enrofloxacin alone	ZJ42-0.75 mg/kg (Total)	0.50	0.25	4	0.13	—	aac (6′)-lb-cr mutant	24 h: 0.40
								48 h: 0.8
	ZJ42-1.25 mg/kg (Total)	0.50	0.50	4	0.13	—	aac (6′)-lb-cr mutant	24 h: 1.38
								48 h: 1.35
	ZJ42-1.5 mg/kg (Split-12 h)	1	1	8	0.13	—	aac (6′)-lb-cr mutant	24 h: 1.61
								48 h: 1.77
cefquinome alone	ZJ42-4 mg/kg (Split-8 h)	0.13	0.13	—	0.5	4	—	—
cefquinome combine	CLS2-1 mg/kg (Split-12 h)	8	8	4	1	8	—	—

### PK/PD modeling of cefquinome in combination with enrofloxacin against *K*. *pneumoniae*

We conducted an *in vitro* competition experiment between the originally sensitive strain and three strains of drug-resistant bacteria induced in the enrofloxacin alone *in vitro* PK/PD model experiment. The relative fitness value (W) of the drug-resistant bacteria at a dose of 0.75 mg/kg was <1 while the W values of the other two strains at doses of 1.25 and 1.5 mg/kg were >1. This indicated that the growth of the latter two strains was not affected by drug resistance mutations except for the drug-resistant bacteria at 0.75 mg/kg (Table 3). We selected the resistant bacteria (ZJ42-1.5s) produced at 30 h when the split dose was 1.5 mg/kg for subsequent *in vitro* PK/PD model studies with combination therapy.

We designed five split doses of cefquinome for the ZJ42-1.5 s and CLS2 strains and kept enrofloxacin constant at 1 × MIC. The cefquinome split dose group alone did not show bactericidal effects for either strain indicating that the bacterial reduction was <3 log_10_ CFU/mL. The split dose groups of cefquinome and enrofloxacin used in combination showed a reduction of >3 log_10_ CFU/mL for both strains at a dose of 4 mg/L and a reduction of >2 log_10_ CFU/mL for both at ≥ 1.25 mg/L (Figures 3C, D and Figures 4C, D).

The E_max_ relationships for the PK/PD parameters *versus* the antibacterial effect of cefquinome in combination with enrofloxacin against the ZJ42-1.5 s and CLS2 indicated that most closely related to efficacy was %T > MIC (*R* ^2^ = 0.956) (Figure 5). The relationship between antibacterial efficacy and PK/PD parameters was then assessed using the sigmoidal E_max_ model to generate E_0_, E_max_, EC_50_ and the Hill coefficient. The magnitude of %T > MIC required for bacterial killing of 3 log_10_ CFU/mL was 18.04 (Table 4). In the different dosage groups of using cefquinome alone and cefquinome combined with enrofloxacin, we found two strains of resistant bacteria with MICs elevated by 4- and 8-fold against cefquinome. However, due to their low frequency and the complexity of the resistance mechanism to cephalosporins, these two strains of resistant bacteria could not be used as reference data (Table 1).

**TABLE 4:** Estimation of PK/PD parameters derived from the inhibitory sigmoidal E_max_ model.

PK/PD parameter	E_0_ (log_10_ CFU/mL)	EC_50_	E_max_ (log_10_ CFU/mL)	Hill coefficient	R^2^	3 Log_10_ CFU/mL reduction
Enrofloxacin alone						
AUC_24h_/MIC (h)	−3.64	24.00	2.90	5.51	0.917	23.29
C_max_/MIC	−4.60	3.92	3.06	2.09	0.951	3.18
%T > MIC	−5.90	72.47	2.67	4.28	0.936	62.71
Cefquinome alone						
AUC_24h_/MIC (h)	−1.81	6.06	2.25	29.19	0.957	6.28
C_max_/MIC	−1.81	4.01	2.25	10.25	0.948	4.44
%T > MIC	−2.32	46.50	2.32	13.28	0.995	48.66
Cefquinome in combination with enrofloxacin						
AUC_24h_/MIC (h)	−2.98	5.06	2.29	3.82	0.941	4.78
C_max_/MIC	−2.84	2.42	2.25	14.78	0.936	2.54
%T > MIC	−3.35	17.43	2.32	3.41	0.959	18.04

E_max_ is the change in the control group (log_10_ CFU/mL) from 0 h to 24 h; E_0_ is the maximum value of the antibacterial effect; EC_50_ is the corresponding PK/PD, index that produces a 50% reduction of the maximum antibacterial effect; N is the Hill coefficient. The “3 Log_10_ CFU/mL reduction” is the PK/PD, index value calculated after a decrease of 3 Log_10_ CFU/mL in E_max_.

## Discussion

Cephalosporin is widely used for human and animal lung infections caused by *K*. *pneumoniae*. In addition, the frequency of reports of cephalosporin-resistant *K*. *pneumoniae* are also increasing. For instance, one study from 2014 to 2016 on chicken farms in China indicated that these farms were a reservoir of Enterobacteriaceae carrying the *bla*NDM-5 gene (Xiang et al., 2018). The presence of antibiotic resistance genes (ARGs) on plasmids and transferable genetic elements in *K*. *pneumoniae* in most likely connected to the improper use of antibiotics on farms. This has resulted in the emergence of extremely drug-resistant (XDR) strains or super-resistance groups (Navon-Venezia et al., 2017). ARGs in *K*. *pneumoniae* can spread from chickens to humans and the incidence of this has been increasing (Elmonir et al., 2021; Wareth and Neubauer, 2021). Therefore, studies of antibiotics used to treat *K*. *pneumoniae* in animals also deepens our understanding of the evolution of drug resistance and associated treatment effects. The current study utilized the two most common antibiotics used to treat *K*. *pneumoniae* infections in animals. Although avibactam is commonly used for combination therapy with cephalosporins, we chose enrofloxacin as the combination object and found that both have a synergistic or partially synergistic effect on multi-strain *K*. *pneumoniae* with different MICs (Table 1).

In our study, we selectively used different strains of bacteria to investigate the bactericidal effects of antibiotics when used individually and to determine whether combining them could enhance the bactericidal effects after an increase in MIC of the strains. We chose strain ZJ42, which is sensitive to both enrofloxacin and cefquinome, for static and dynamic models. In the *in vitro* dynamic PK/PD model of enrofloxacin against ZJ42, we isolated three derived strains of ZJ42 with increased MIC at 24 or 30 h from the dosing group (Table 3). Through *in vitro* competition tests, we selected one well-growing derived strain isolated from the dosing group at a dose of 1.5 mg/L (Split), designated as ZJ42-1.5s, for combination research. We also selected another strain, CLS2, which was low-level resistant to enrofloxacin but sensitive to cefquinome, as a reference strain. We did not use the ATCC 700603 strain in our experiment because its MIC was higher than the concentrations achievable with enrofloxacin and cefquinome in food animals, and it was not recommended for routine antibiotic treatment in clinical practice (Table 1).

The *in vitro* static bactericidal model of enrofloxacin and cefquinome indicated that the killing curve of enrofloxacin against *K*. *pneumoniae* possessed a steeper slope than that of cefquinome indicating a better bactericidal effect with a bacterial load of ∼10^6^ CFU/mL (Figures 1A, B). The more rapid bactericidal rate of fluoroquinolones compared to β-lactams has been previously reported where the time required for fluoroquinolones to produce bactericidal effects was 1.5 h for *E*. *coli*, 4–6 h for and ≥6 h for streptococci (Fung-Tomc et al., 2000). We chose a concentration range of 0–8 MIC for our experiments and for each level, tolerance was not increased as the concentration was increased. We also applied the sigmoid E_max_ model that can comprehensively assess growth and killing rates of bacteria in the presence of drugs to provide guidance for optimizing dosing regimens (Nielsen et al., 2007; Zhang et al., 2018a). We chose the 0–9 h window because after 9 h, CFU differences were not obvious on the curve. The simulation fitting results from 0–9 h were good with *R* ^2^ values of 0.99, 0.996, 0.986, and 1.000 for the four single and combined drug use curves. The combined drug use displayed the highest Emax (strain ZJ42-1.5s) and cefquinome alone has the smallest Emax (ZJ42) indicating that combined use could improve the antibacterial effect (Table 2).

We adopted a one-compartment open model to simulate the PK of chickens to study the effect of dynamic drug concentrations against *K*. *pneumoniae*. Due to the rapid absorption of enrofloxacin and cefquinome following extravascular administration, absorption was not considered in this model. We selected two bacterial strains with different sensitivities to enrofloxacin to ensure robust experimental results. For both strains, we designed separate dosing groups for monotherapy and combination therapy to visually demonstrate the differences in the antibacterial effects of different dosing regimens. To reflect the pharmacokinetic rules and states of enrofloxacin and cefquinome in the chicken body, we set up multiple dosing groups based on the pharmacokinetic parameters of the drugs in the chicken body when enrofloxacin was orally administered at a dose of 10 mg/kg and cefquinome was intramuscularly injected at a dose of 2 mg/kg (Xie et al., 2013; Atef et al., 2020). Although immune factors in animals have not been considered, studies using *in vitro* models have allowed examination of a direct relationship between dosing regimens and bacteriological effects. The *in vitro* dynamic time–kill curve was a preliminary characterization of the activity of the drug. We then fitted the data to the sigmoidal E_max_ model to obtain specific PK/PD parameters. The parameter value corresponding to a certain antibacterial effect was calculated by the PK/PD index that was obtained. Furthermore, PK/PD interactions were analyzed to determine the dependence of the concentration and/or time of the activities (Zhang H et al., 2019).

Antibiotics can also be divided into time- and concentration-dependent drugs (Zhang L et al., 2022). For concentration-dependent drugs, reductions in bacterial survival are positively correlated with drug concentration. In the *in vitro* static model of our experiments, enrofloxacin showed concentration dependence at 0–8 × MIC while the performance of cefquinome tended to be time-dependent (Figures 1A, B). At some concentrations, *K*. *pneumoniae* levels may recover after a decline suggesting two possible reasons: one is the emergence of drug-resistance mutations that may be transient or long-term. The other is the degradation of the drug in interaction with bacteria and culture media and this has been previously reported for *Actinobacillus pleuropneumoniae* and *Riemerella anatipestifer* (Zhang et al., 2018b; Zhang L et al., 2019). In our study, further research is needed to determine between the exact cause for the recovery. Since the CFU counts were below the detection limit after 9 h at > 4 × MIC or 0.5 mg/L, dynamic PK/PD model studies should be conducted.

The *in vitro* dynamic model study of enrofloxacin indicated that at ≥1.5 mg/L, enrofloxacin caused CFU reductions for strain ZJ42 of ≥4.45 log_10_ CFU/mL. The correlation coefficients of AUC_24 h_/MIC, C_max_/MIC, and %T > MIC with an antibacterial effect were 0.917, 0.951, and 0.936, respectively (Table 3). In the time-kill curve of the dynamic model, the relationship between drug concentration and CFU changed proportionally. The fitting curve of AUC_24 h_/MIC and C_max_/MIC in PK/PD was more reasonable while the decreasing trend in the fitting curve of %T > MIC did not conform to a pharmacological effect on the bacteria. This indicated that the enrofloxacin effects were concentration-dependent with C_max_ of 0.2–2 mg/L. Therefore, we selected the fitting results between data of parameters AUC_24 h_/MIC and C_max_/MIC along with changes in bacterial CFU numbers. The AUC_24h_/MIC and C_max_/MIC values, which caused a decrease of 3 log_10_ CFU/mL when enrofloxacin was used alone, were 23.29h and 3.18, respectively (Table 4).

For strains not sensitive to enrofloxacin alone, we used cefquinome alone and combined with enrofloxacin at a constant concentration of 1× MIC to conduct a comparative analysis. Cefquinome alone did not exhibit bactericidal activity against ZJ42, ZJ42-1.5s or CLS2 at a split dosage of 1.5–4 mg/L within 0–24 h and bacterial growth was not reduced by > 3 log_10_ CFU/mL. However, when cefquinome was used in combination with enrofloxacin at 1 × MIC, bactericidal activity against ZJ42-1.5s and CLS2 was observed when the 12 h split dose was ≥4 mg/L and bacterial growth was reduced by ≥ 3.65 log_10_ CFU/mL. Cefquinome used alone generated an antibacterial effect for an 8 h split dose that was better than that of a 12 h split dose. However, considering the convenience of clinical application, we prefer to use a 12 h split dose in subsequent studies.

In the dynamic model of single-dose enrofloxacin at a constant dose of 1 ×MIC, the growth of *K*. *pneumoniae* was not affected and this differed from the static model. The reason may be that in the dynamic model, *K*. *pneumoniae* survives better at a constant concentration of antibiotics. When the dose of cefquinome was at C_max_ of 2 and 4 mg/L in combination therapy, its antibacterial effect was superior to that of using cefquinome alone (Figures 4C, D). The correlation coefficients of AUC_24 h_/MIC, C_max_/MIC, and %T > MIC for cefquinome alone *versus* combination therapy were 0.957 vs. 0.941, 0.948 vs. 0.936 and 0.995 vs. 0.959, respectively. The correlation between drug concentration and bacterial load was not significant when cefquinome was used alone or in combination with enrofloxacin. In the fitting results of the inhibitory sigmoidal E_max_ model, the fitting curve of %T > MIC was the most reasonable while the fitting curves for AUC_24 h_/MIC and C_max_/MIC displayed decreasing trends that did not comply with the law of drug killing. In the fitted curves of AUC_24h_/MIC and C_max_/MIC, the values of AUC_24h_/MIC and C_max_/MIC did not change when the ΔE increased (Figure 5 B1, B2, C1, and C2). The above results indicated that cefquinome exhibited time-dependent effects against *K*. *pneumoniae* and was driven by %T > MIC. The %T > MIC required to cause a 3 log_10_ CFU/mL reduction for cefquinome used alone and in combination were 48.66 and 18.04 respectively. Hence, combination therapy of enrofloxacin and cefquinome with a high MIC was efficacious against the infections caused by *K*. *pneumoniae*. Possibly, the presence of enrofloxacin reduced the MIC of cefquinome and thereby improved the %T > MIC.

Previous studies have indicated that enrofloxacin exhibits concentration-dependent killing of respiratory pathogens in animals and is driven by AUC_24h_/MIC and C_max_/MIC (Intorre et al., 2000). In our study, a comparison of single-dose and split-dose administration was conducted for enrofloxacin and we found that increasing %T > MIC by split-dose administration did not significantly improve the bactericidal effect. A previous reports had indicated that the AUC_24h_/MIC of enrofloxacin for a 3 log_10_ CFU/mL reduction caused by *E*. *coli* was 23.41 h and the highest correlation coefficient of the fitted AUC_24h_/MIC was 0.91 (Xiao et al., 2018). This indicated a long T_1/β_ of 18.77 h for enrofloxacin. In cases where T_1/β_ is long, it is more reasonable to use AUC_24h_/MIC to predict the dosing regimen of enrofloxacin. In our study, T_1/β_ was set to 6.53 h and the fitting coefficient of C_max_/MIC was higher than that of AUC_24h_/MIC. Therefore, perhaps using f C_max_/MIC to predict the therapeutic outcome of enrofloxacin for drug dynamic parameters with shorter T_1/β_ may be a more feasible approach.

Research on cefquinome against Gram-negative bacteria has shown that if the T_1/β_ dosing interval is relatively short, f T > MIC can be used for prediction. If the T_1/β_ dosing interval is relatively long, f AUC/MIC is used (Kristoffersson et al., 2016). In our study, we found that a contradiction between the shorter T_1/β_ of cefquinome and the strong survival ability of *K*. *pneumoniae* that resulted in poor therapeutic efficacy when cefquinome was used alone. Shortening the dosing interval of cefquinome and using a higher initial concentration did not reduce the bacterial CFU counts to below detection limits and it quickly rebounded after the drug concentration fell below the MIC (Figure 4B). When enrofloxacin was used in combination with a constant 1× MIC, this situation improved significantly and %T > MIC decreased from 48.66 to 18.04 (Table 4). When treating lung infections caused by *K*. *pneumoniae* in chickens with cefquinome, it is difficult to shorten the dosing interval. In this case, using an enrofloxacin and cefquinome combination and predicting the dosing regimen based on f T > MIC would be a good choice.

After analyzing the changes in bacterial susceptibility in the *in vitro* dynamic model at 24 or 30 h, we found that the use of enrofloxacin was more likely to produce resistant bacteria compared to the use of cefquinome with a 4-8 fold increase in MIC of resistant bacteria (Table 3). A comparison of the resistance genes of the original susceptible bacteria and induced resistant bacteria revealed that the low-level increase in bacterial MIC was due to mutations in the *aac* (6′)-lb-cr (Table 3). This was consistent with previous studies that reported a moderate increase in MIC of affected quinolone drugs caused by *aac* (6′)-lb-cr (Park et al., 2006; Guillard et al., 2013; Rodríguez-Martínez et al., 2016). No resistance mutations were found in the QRDR nor were other resistance plasmids found. Therefore, *aac* (6’)-lb-cr may be more likely to cause low-level resistance of *K*. *pneumoniae* to enrofloxacin than QRDRs but further research is needed to verify this conclusion.

The *in vitro* static and dynamic PK/PD models had the advantages of convenience and intuitiveness. We could understand the effects and patterns of drug action on bacteria in a controllable environment, providing a reference for the *in vivo* PK/PD research. However, the *in vitro* PK/PD studies had limitations. After referring to relevant the *in vitro* PK/PD studies, we used MHII broth as the bacterial culture medium (Kristoffersson et al., 2020). This medium did not contain plasma or albumin, resulting in a lower protein binding rate compared to plasma protein binding rate, was an important factor to consider when interpreting the results of our *in vitro* PK/PD models. According to reports, enrofloxacin had a higher binding rate in animal plasma, ranging from 31% to 46% (Bidgood and Papich, 2005; Davis et al., 2007; Messenger et al., 2012), while cefquinome had a lower binding rate in animal plasma, at 16.4% (Zhang L et al., 2019). Therefore, this difference might potentially lead to variations in drug behavior and affect the accuracy of our findings. We based on the plasma protein binding rates of enrofloxacin and cefquinome referred to the literature, and according to the pharmacokinetic parameters of enrofloxacin oral administration and cefquinome intramuscular injection in chicken bodies reported in the literature (Xie et al., 2013; Xiao et al., 2018). According to the derivation formula for the best dosing regimen (Toutain et al., 2007), the doses of enrofloxacin and cefquinome that reduction the bacterial count of ZJ42 by 3 Log_10_ CFU/mL were 2.51 and 3.96 mg/kg per day, respectively. Considering the pharmacokinetic parameters of animals of different breeds or ages might was different, the recommended drug regimen needs to be determined through the *in vivo* PK/PD experiments. Since the number of induced cefquinome - resistant strains of was small and only one strain was induced in each, these data could not be used as a reference. However, these small numbers may be due to a combination of enrofloxacin at 1 × MIC and the short T_1/β_ of cefquinome that did not lead to resistance development. This phenomenon shows some advantages and in the *in vitro* dynamic model selective pressure comes only from antibiotic concentration and two-thirds of the competitive growth capacity of resistant bacteria was equivalent to that of sensitive bacteria (Table 3). Bacteria will grow unrestrictedly at < 1× MIC.

## Conclusion

Enrofloxacin and cefquinome displayed synergistic and additive effects against *K*. *pneumoniae* in the checkerboard assay. Enrofloxacin alone exhibited significant concentration-dependent features while cefquinome alone or in combination with enrofloxacin exhibited significant time-dependent features in their antibacterial effects against *K*. *pneumoniae*. The AUC_24h_/MIC and C_max_/MIC parameters of enrofloxacin for reducing 3 log_10_ CFU/mL were determined to be 23.29 h and 3.18, respectively. The antibacterial effect of cefquinome against *K*. *pneumoniae* was enhanced when it was used in combination with enrofloxacin. When used alone, the % T > MIC parameter for cefquinome to reduce 3 log_10_ CFU/mL was 48.66 and this was decreased to 18.04 when used in combination with enrofloxacin. In treating lung infections caused by sensitive *K*. *pneumoniae*, enrofloxacin alone exhibited a better antibacterial effect than cefquinome alone. However, in treating lung disease caused by *K*. *pneumoniae*, the antibacterial effect of cefquinome can be enhanced by combining it with enrofloxacin. These findings suggest a potentially effective approach for combating *K*. *pneumoniae* infections.

## Data Availability

The original contributions presented in the study are included in the article/Supplementary Material, further inquiries can be directed to the corresponding author.
