# Supplementary material for: PK-PD integration of enrofloxacin and cefquinome alone and in combination against Klebsiella pneumoniae using an in vitro dynamic model
Source: Front Pharmacol. 2023 Oct 6;14:1226936. doi: 10.3389/fphar.2023.1226936 (PMC10587432; doi:10.3389/fphar.2023.1226936)
Supplement: Supplementary file 1 [file DataSheet1.ZIP › Chromatogram/enrofloxacin/0.25 0.75ppm/1000tj.pdf]

==

操作者 : 系统　　序列行 : 10
仪器 : 1260　　位置 : P1-A10
进样日期 : 2022/12/13 22:27:08　　进样次数 : 1
进样量 : 50.000 µl
采集方法 : D:\1260\data\wyz2022\WYZ-ENR22.12.12 2022-12-13 20-15-53\wyz 2020.07.6bayer2BH.M
最后修改 : 2022/12/13 20:42:52 : 系统
分析方法 : D:\1260\data\wyz2022\WYZ-ENR22.12.12 2022-12-13 20-15-53\wyz 2020.07.6bayer2BH.M (序列方法)
最后修改 : 2022/12/14 11:44:32 : 系统
(调用后修改)
附加信息：峰被手动积分

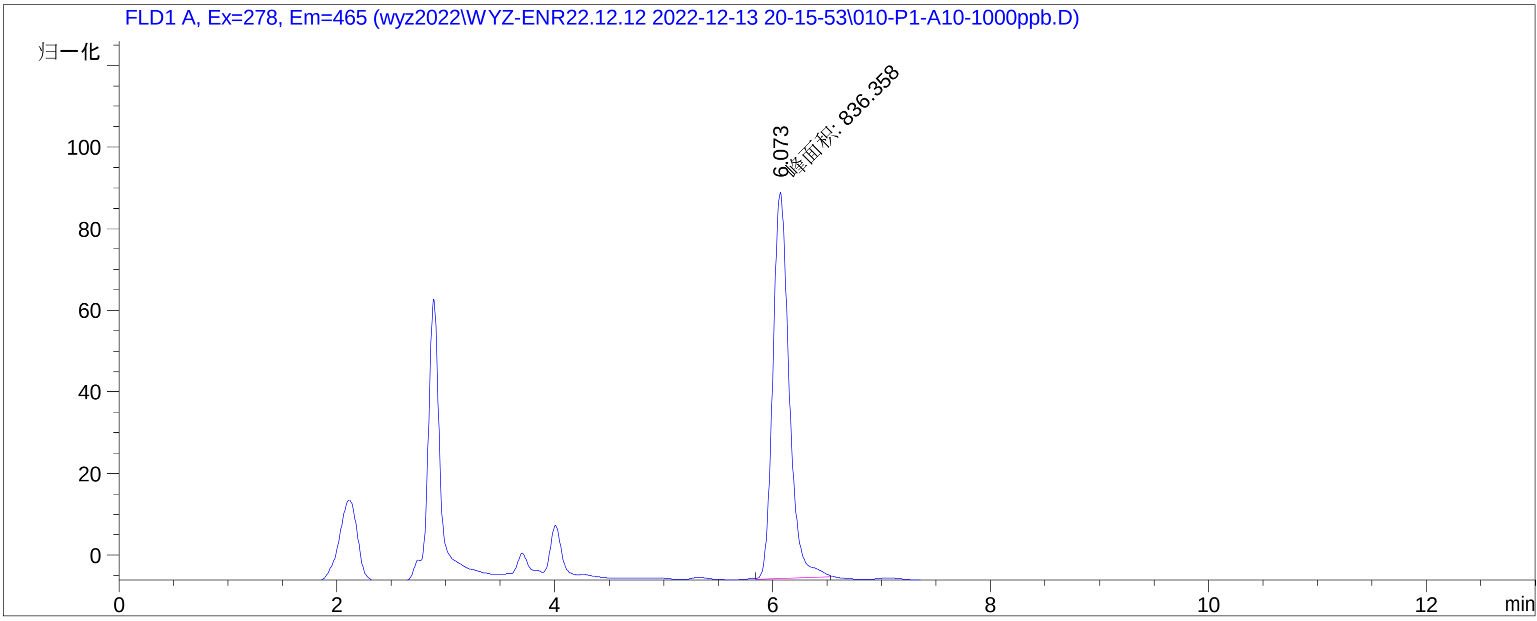

==

面积百分比报告

==

排序 : 信号
乘积因子 : 1.0000
稀释因子 : 1.0000
内标中不使用乘积因子和稀释因子

信号 1: FLD1 A, Ex=278, Em=465

峰 #	保留时间 [min]	类型	峰宽 [min]	峰面积 [LU*s]	峰高 [LU]	峰面积 %
1	6.073	MM	0.1699	836.35754	82.04774	100.0000

总量 : 836.35754　82.04774

==

*** 报告结束 ***
