# Supplementary material for: PK-PD integration of enrofloxacin and cefquinome alone and in combination against Klebsiella pneumoniae using an in vitro dynamic model
Source: Front Pharmacol. 2023 Oct 6;14:1226936. doi: 10.3389/fphar.2023.1226936 (PMC10587432; doi:10.3389/fphar.2023.1226936)
Supplement: Supplementary file 1 [file DataSheet1.ZIP › Chromatogram/enrofloxacin/0.25 0.75ppm/50tj.pdf]

==

操作者	: 系统	序列行 :	6
仪器	: 1260	位置 :	P1-A6
进样日期	: 2022/12/13 21:30:03	进样次数 :	1
		进样量 :	50.000 µl

采集方法　　: D:\1260\data\wyz2022\WYZ-ENR22.12.12 2022-12-13 20-15-53\wyz 2020.07.6bayer2BH.M
最后修改　　: 2022/12/13 20:42:52 :　系统
分析方法　　: D:\1260\data\wyz2022\WYZ-ENR22.12.12 2022-12-13 20-15-53\wyz 2020.07.6bayer2BH.M (序列方法)
最后修改　　: 2022/12/14 11:42:26 :　系统
(调用后修改)
附加信息：峰被手动积分

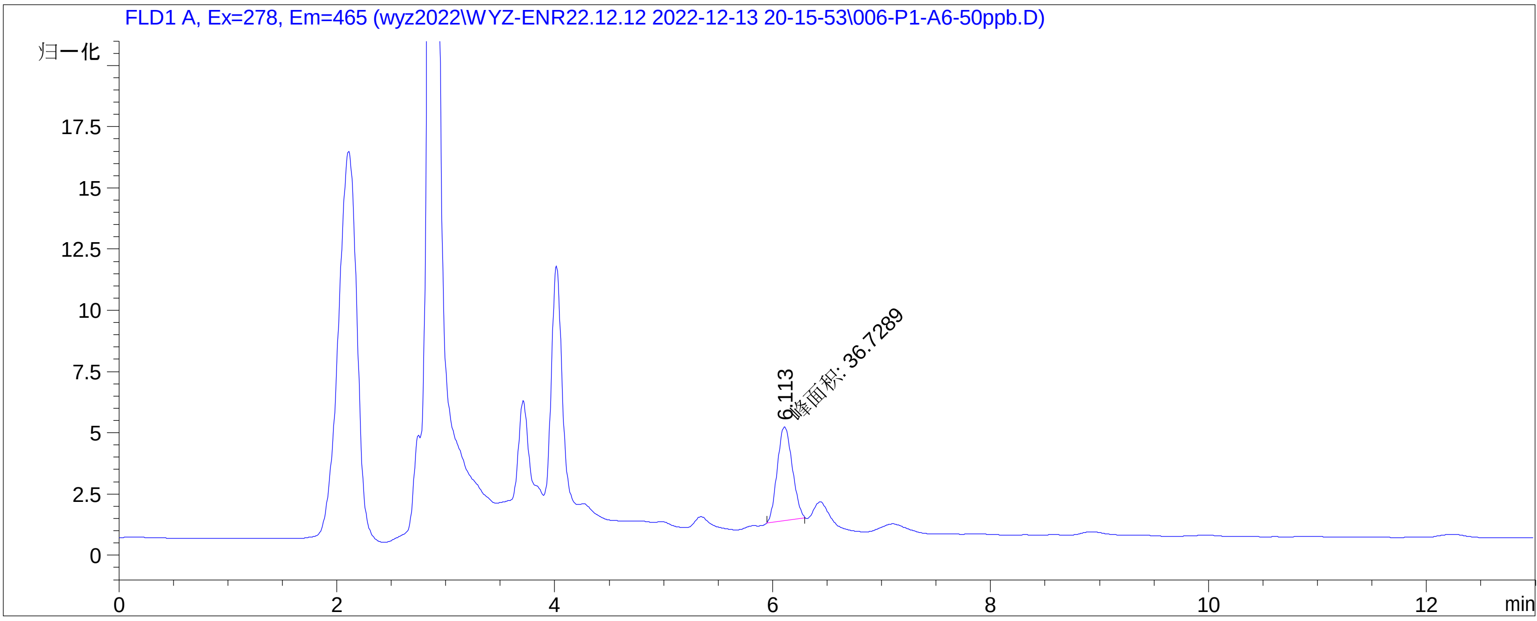

==
面积百分比报告
==

排序　　　　　:　信号
乘积因子　　　:　1.0000
稀释因子　　　:　1.0000
内标中不使用乘积因子和稀释因子

信号 1: FLD1 A, Ex=278, Em=465

峰 #	保留时间 [min]	类型	峰宽 [min]	峰面积 [LU*s]	峰高 [LU]	峰面积 %
1	6.113	MM	0.1586	36.72891	3.85868	100.0000

总量 :　36.72891　3.85868

==
*** 报告结束 ***
