# Supplementary material for: PK-PD integration of enrofloxacin and cefquinome alone and in combination against Klebsiella pneumoniae using an in vitro dynamic model
Source: Front Pharmacol. 2023 Oct 6;14:1226936. doi: 10.3389/fphar.2023.1226936 (PMC10587432; doi:10.3389/fphar.2023.1226936)
Supplement: Supplementary file 1 [file DataSheet1.ZIP › Chromatogram/enrofloxacin/1.25 1.5ppm/1.5T-30H.pdf]

数据文件: D:\1260\data\wyz2022\WYZ-ENR22.12.12 2022-12-14 19-40-20\033-P1-C8-1.5T-30.D
样品名称: 1.5T-30

=====================================================================

| | | | |
|---|---|---|---|
| 操作者 | : 系统 | 序列行 : | 33 |
| 仪器 | : 1260 | 位置 : | P1-C8 |
| 进样日期 | : 2022/12/15 3:16:52 | 进样次数 : | 1 |
| | | 进样量 : | 50.000 µl |

采集方法 : D:\1260\data\wyz2022\WYZ-ENR22.12.12 2022-12-14 19-40-20\wyz 2020.07.6bayer2BH.M
最后修改 : 2022/12/14 19:45:05 : 系统
分析方法 : D:\1260\data\wyz2022\WYZ-ENR22.12.12 2022-12-14 19-40-20\wyz 2020.07.6bayer2BH.M (序列方法)
最后修改 : 2022/12/15 11:11:57 : 系统
(调用后修改)
附加信息：峰被手动积分

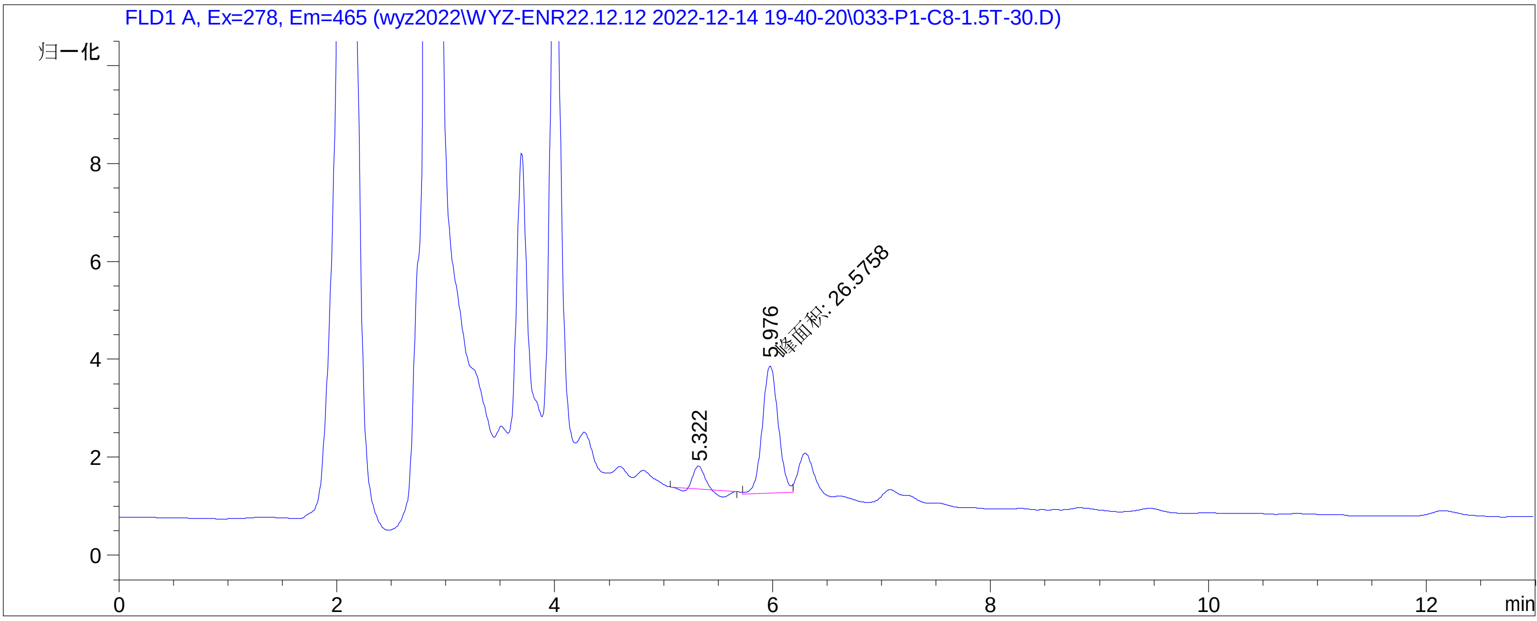


=====================================================================
面积百分比报告
=====================================================================

排序 : 信号
乘积因子 : 1.0000
稀释因子 : 1.0000
内标中不使用乘积因子和稀释因子

信号 1: FLD1 A, Ex=278, Em=465

| 峰 # | 保留时间 [min] | 类型 | 峰宽 [min] | 峰面积 [LU*s] | 峰高 [LU] | 峰面积 % |
|---|---|---|---|---|---|---|
| 1 | 5.322 | BB | 0.1054 | 2.02972 | 4.42038e-1 | 7.0955 |
| 2 | 5.976 | MM | 0.1672 | 26.57583 | 2.64902 | 92.9045 |

总量 : 28.60555 3.09106

=====================================================================
*** 报告结束 ***
