# Supplementary material for: PK-PD integration of enrofloxacin and cefquinome alone and in combination against Klebsiella pneumoniae using an in vitro dynamic model
Source: Front Pharmacol. 2023 Oct 6;14:1226936. doi: 10.3389/fphar.2023.1226936 (PMC10587432; doi:10.3389/fphar.2023.1226936)
Supplement: Supplementary file 1 [file DataSheet1.ZIP › Chromatogram/enrofloxacin/1.25 1.5ppm/1.75T-1H.pdf]

数据文件: D:\1260\data\wyz2022\WYZ-ENR22.12.12 2022-12-14 19-40-20\036-P1-C10-1.75T-1.D
样品名称: 1.75T-1

===

操作者 : 系统　　　　序列行 : 36
仪器 : 1260　　　　位置 : P1-C10
进样日期 : 2022/12/15 3:59:14　　　　进样次数 : 1
进样量 : 50.000 µl

采集方法 : D:\1260\data\wyz2022\WYZ-ENR22.12.12 2022-12-14 19-40-20\wyz 2020.07.6bayer2BH.M
最后修改 : 2022/12/14 19:45:05 : 系统
分析方法 : D:\1260\data\wyz2022\WYZ-ENR22.12.12 2022-12-14 19-40-20\wyz 2020.07.6bayer2BH.M (序列方法)
最后修改 : 2022/12/15 11:14:36 : 系统
(调用后修改)
附加信息：峰被手动积分

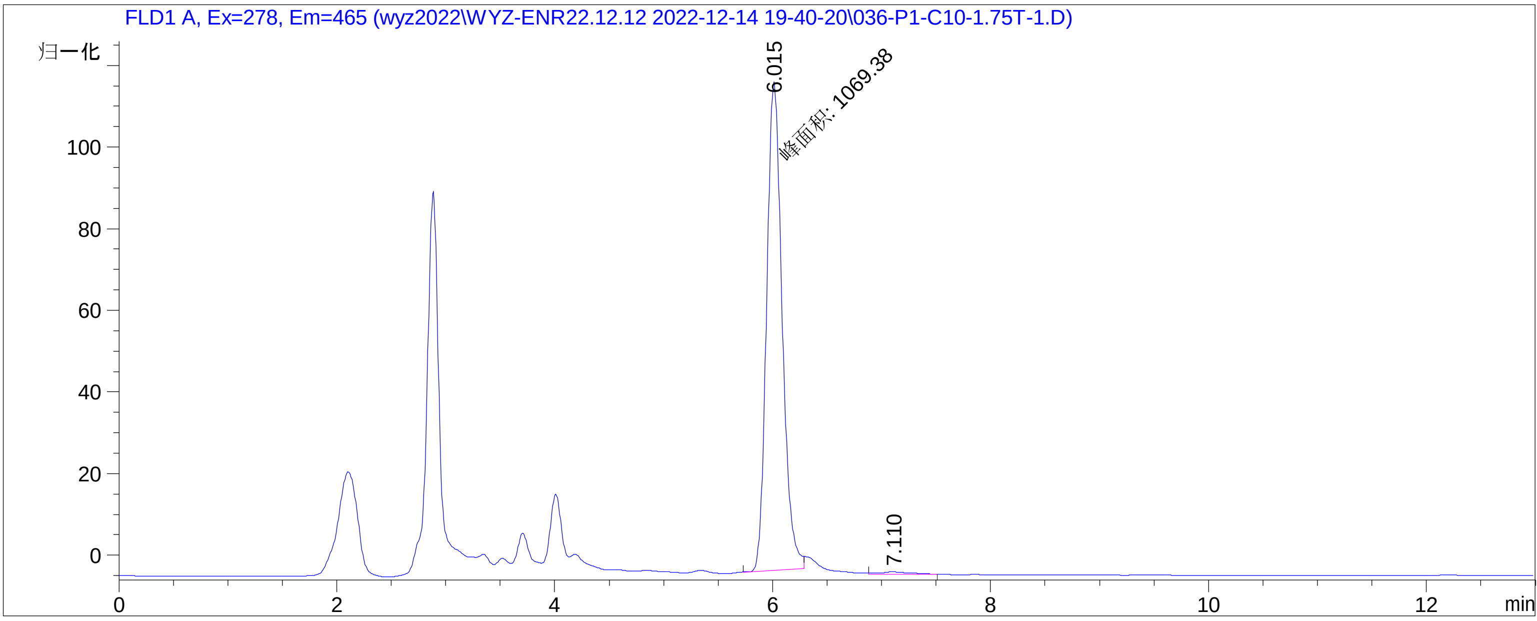

===

面积百分比报告

===

排序 : 信号
乘积因子 : 1.0000
稀释因子 : 1.0000
内标中不使用乘积因子和稀释因子

信号 1: FLD1 A, Ex=278, Em=465

峰 #	保留时间 [min]	类型	峰宽 [min]	峰面积 [LU*s]	峰高 [LU]	峰面积 %
1	6.015	MM	0.1618	1069.37805	110.14745	99.0362
2	7.110	BB	0.2869	10.40703	5.16522e-1	0.9638

总量 : 1079.78508 110.66398

===

*** 报告结束 ***
