# Supplementary material for: PK-PD integration of enrofloxacin and cefquinome alone and in combination against Klebsiella pneumoniae using an in vitro dynamic model
Source: Front Pharmacol. 2023 Oct 6;14:1226936. doi: 10.3389/fphar.2023.1226936 (PMC10587432; doi:10.3389/fphar.2023.1226936)
Supplement: Supplementary file 1 [file DataSheet1.ZIP › Chromatogram/enrofloxacin/1.25 1.5ppm/1.75T-30H.pdf]

数据文件: D:\1260\data\wyz2022\WYZ-ENR22.12.12 2022-12-14 19-40-20\042-P1-D5-1.75T-30.D
样品名称: 1.75T-30

===

操作者	: 系统	序列行 :	42
仪器	: 1260	位置 :	P1-D5
进样日期	: 2022/12/15 5:24:53	进样次数 :	1
		进样量 :	50.000 µl

采集方法　　: D:\1260\data\wyz2022\WYZ-ENR22.12.12 2022-12-14 19-40-20\wyz 2020.07.6bayer2BH.M
最后修改　　: 2022/12/14 19:45:05 : 系统
分析方法　　: D:\1260\data\wyz2022\WYZ-ENR22.12.12 2022-12-14 19-40-20\wyz 2020.07.6bayer2BH.M (序列方法)
最后修改　　: 2022/12/15 11:18:33 : 系统
(调用后修改)
附加信息：峰被手动积分

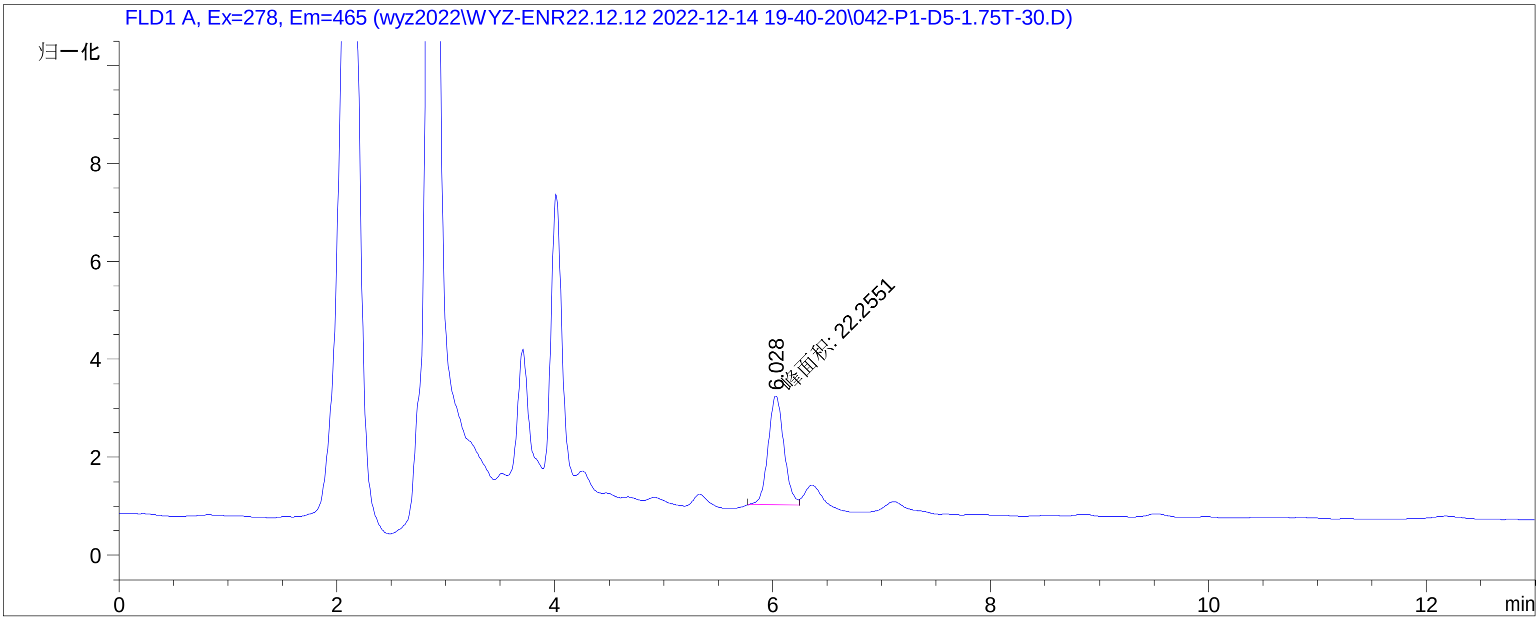

===

面积百分比报告

===

排序　　　　　　　: 信号
乘积因子　　　　　: 1.0000
稀释因子　　　　　: 1.0000
内标中不使用乘积因子和稀释因子

信号 1: FLD1 A, Ex=278, Em=465

峰 #	保留时间 [min]	类型	峰宽 [min]	峰面积 [LU*s]	峰高 [LU]	峰面积 %
1	6.028	MM	0.1674	22.25508	2.21552	100.0000

总量 :　22.25508　2.21552

===

*** 报告结束 ***
