# Supplementary material for: PK-PD integration of enrofloxacin and cefquinome alone and in combination against Klebsiella pneumoniae using an in vitro dynamic model
Source: Front Pharmacol. 2023 Oct 6;14:1226936. doi: 10.3389/fphar.2023.1226936 (PMC10587432; doi:10.3389/fphar.2023.1226936)
Supplement: Supplementary file 1 [file DataSheet1.ZIP › Chromatogram/enrofloxacin/1S 1.5S 2S 2Tppm/2S ppmRetest/ENR2S-0.pdf]

数据文件: D:\1260\data\wyz2022\WYZ-ENR22.12.12 2022-12-17 17-50-35\005-P1-D5-ENR2S-0.D
样品名称: ENR2S-0

=====================================================================

操作者 : 系统 序列行 : 5
仪器 : 1260 位置 : P1-D5
进样日期 : 2022/12/17 18:49:09 进样次数 : 1
进样量 : 50.000 μl
采集方法 : D:\1260\data\wyz2022\WYZ-ENR22.12.12 2022-12-17 17-50-35\wyz 2020.08.6bayer2BH.M
最后修改 : 2022/12/17 17:50:35 : 系统
分析方法 : D:\1260\data\wyz2022\WYZ-ENR22.12.12 2022-12-17 17-50-35\wyz 2020.08.6bayer2BH.M (序列方法)
最后修改 : 2022/12/18 19:11:08 : 系统
(调用后修改)
附加信息：峰被手动积分

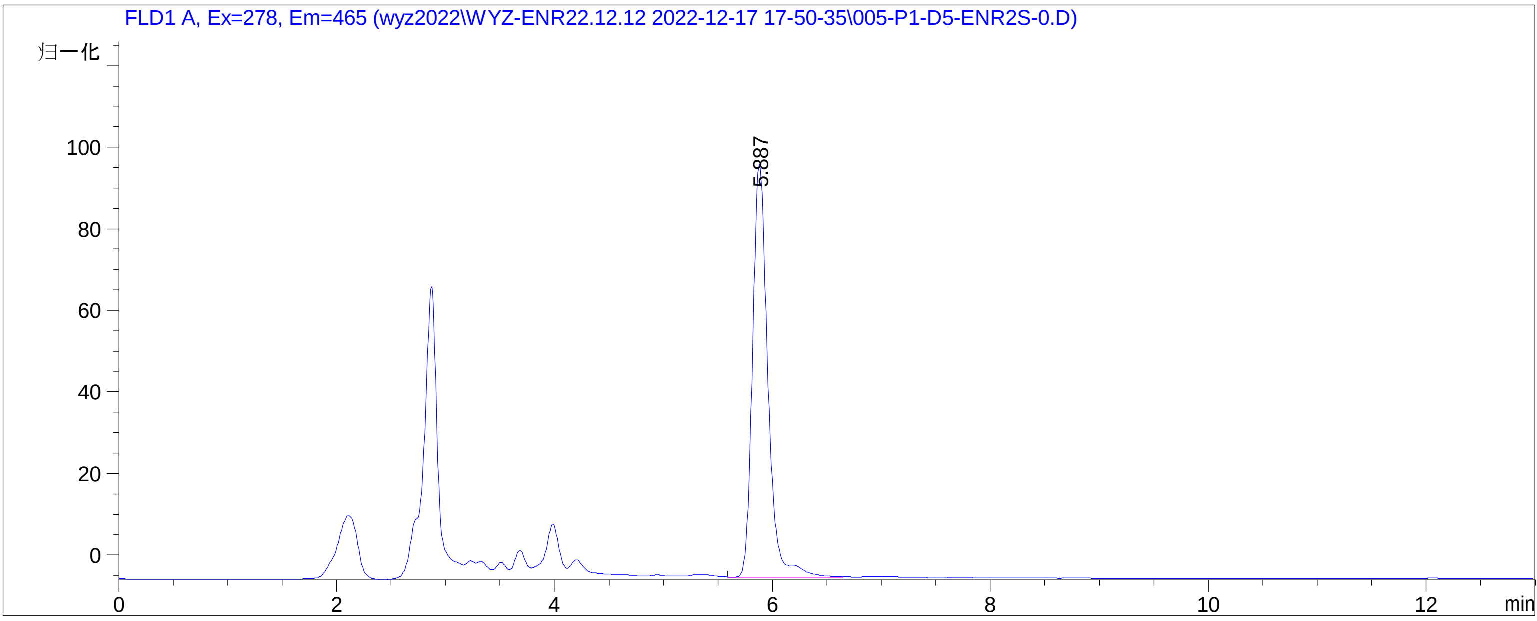


=====================================================================

面积百分比报告

=====================================================================

排序 : 信号
乘积因子 : 1.0000
稀释因子 : 1.0000
内标中不使用乘积因子和稀释因子

信号 1: FLD1 A, Ex=278, Em=465

| 峰 # | 保留时间 [min] | 类型 | 峰宽 [min] | 峰面积 [LU*s] | 峰高 [LU] | 峰面积 % |
|---|---|---|---|---|---|---|
| 1 | 5.887 | VB | 0.1753 | 861.69537 | 81.41520 | 100.0000 |

总量 : 861.69537 81.41520

=====================================================================

*** 报告结束 ***
