# Supplementary material for: PK-PD integration of enrofloxacin and cefquinome alone and in combination against Klebsiella pneumoniae using an in vitro dynamic model
Source: Front Pharmacol. 2023 Oct 6;14:1226936. doi: 10.3389/fphar.2023.1226936 (PMC10587432; doi:10.3389/fphar.2023.1226936)
Supplement: Supplementary file 1 [file DataSheet1.ZIP › Chromatogram/enrofloxacin/1S 1.5S 2S 2Tppm/2S ppmRetest/ENR2S-24.pdf]

=====================================================================

| | | | |
|---|---|---|---|
| 操作者 | : 系统 | 序列行 : | 13 |
| 仪器 | : 1260 | 位置 : | P1-E2 |
| 进样日期 | : 2022/12/17 20:43:42 | 进样次数 : | 1 |
| | | 进样量 : | 50.000 µl |

采集方法 : D:\1260\data\wyz2022\WYZ-ENR22.12.12 2022-12-17 17-50-35\wyz 2020.08.6bayer2BH.M
最后修改 : 2022/12/17 17:50:35 : 系统
分析方法 : D:\1260\data\wyz2022\WYZ-ENR22.12.12 2022-12-17 17-50-35\wyz 2020.08.6bayer2BH.M (序列方法)
最后修改 : 2022/12/18 19:21:32 : 系统
(调用后修改)
附加信息：峰被手动积分

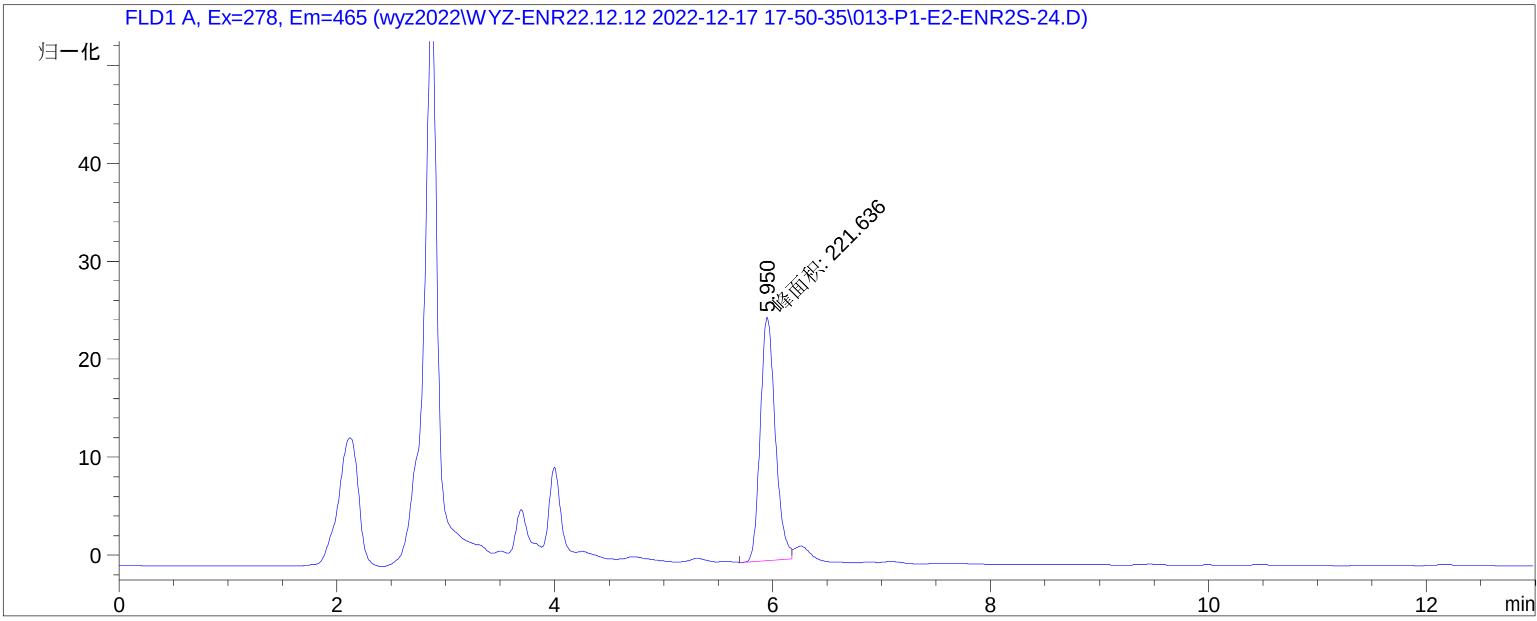


=====================================================================

面积百分比报告

=====================================================================

排序 : 信号
乘积因子 : 1.0000
稀释因子 : 1.0000
内标中不使用乘积因子和稀释因子

信号 1: FLD1 A, Ex=278, Em=465

| 峰 # | 保留时间 [min] | 类型 | 峰宽 [min] | 峰面积 [LU*s] | 峰高 [LU] | 峰面积 % |
|---|---|---|---|---|---|---|
| 1 | 5.950 | MM | 0.1604 | 221.63641 | 23.02819 | 100.0000 |

总量 : 221.63641 23.02819

=====================================================================

*** 报告结束 ***
