# Supplementary material for: PK-PD integration of enrofloxacin and cefquinome alone and in combination against Klebsiella pneumoniae using an in vitro dynamic model
Source: Front Pharmacol. 2023 Oct 6;14:1226936. doi: 10.3389/fphar.2023.1226936 (PMC10587432; doi:10.3389/fphar.2023.1226936)
Supplement: Supplementary file 1 [file DataSheet1.ZIP › Chromatogram/enrofloxacin/1S 1.5S 2S 2Tppm/50ppb.pdf]

数据文件: D:\1260\data\wyz2022\WYZ-ENR22.12.12 2022-12-16 12-12-30\001-P1-A3-50ppb.D
样品名称: 50ppb

===

操作者	: 系统	序列行 :	1
仪器	: 1260	位置 :	P1-A3
进样日期	: 2022/12/16 12:13:29	进样次数 :	1
		进样量 :	50.000 µl

采集方法 : D:\1260\data\wyz2022\WYZ-ENR22.12.12 2022-12-16 12-12-30\wyz 2020.07.6bayer2BH.M
最后修改 : 2022/12/16 12:15:58 : 系统
(调用后修改)
分析方法 : D:\1260\data\wyz2022\WYZ-ENR22.12.12 2022-12-16 12-12-30\wyz 2020.07.6bayer2BH.M (序列方法)
最后修改 : 2022/12/17 11:20:53 : 系统
(调用后修改)
附加信息：峰被手动积分

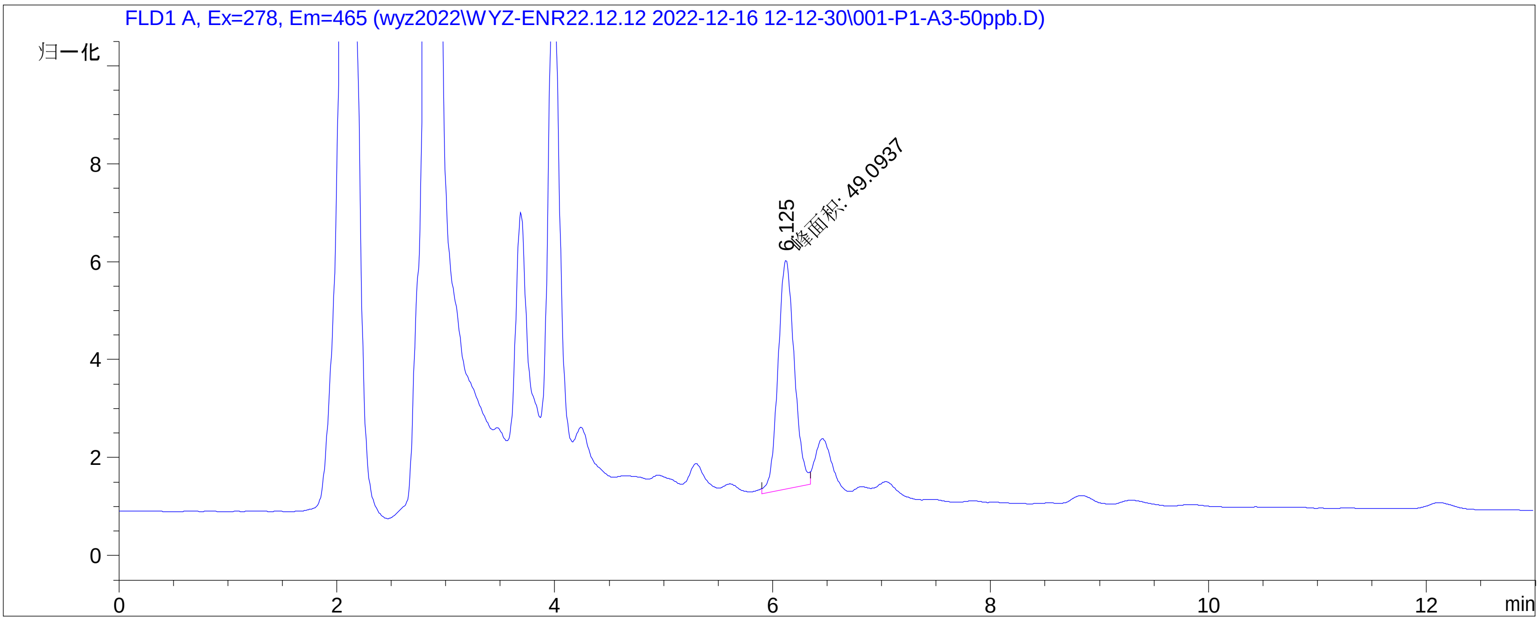

===

面积百分比报告

===

排序 : 信号
乘积因子 : 1.0000
稀释因子 : 1.0000
内标中不使用乘积因子和稀释因子

信号 1: FLD1 A, Ex=278, Em=465

峰 #	保留时间 [min]	类型	峰宽 [min]	峰面积 [LU*s]	峰高 [LU]	峰面积 %
1	6.125	MM	0.1726	49.09367	4.73977	100.0000

总量 : 49.09367 4.73977

===

*** 报告结束 ***
