# Supplementary material for: PK-PD integration of enrofloxacin and cefquinome alone and in combination against Klebsiella pneumoniae using an in vitro dynamic model
Source: Front Pharmacol. 2023 Oct 6;14:1226936. doi: 10.3389/fphar.2023.1226936 (PMC10587432; doi:10.3389/fphar.2023.1226936)
Supplement: Supplementary file 1 [file DataSheet1.ZIP › Chromatogram/enrofloxacin/1S 1.5S 2S 2Tppm/ENR1S-3.pdf]

数据文件: D:\1260\data\wyz2022\WYZ-ENR22.12.12 2022-12-16 12-12-30\010-P1-A11-ENR1S-3.D
样品名称: ENR1S-3

===

操作者	: 系统	序列行 :	10
仪器	: 1260	位置 :	P1-A11
进样日期	: 2022/12/16 14:21:29	进样次数 :	1
		进样量 :	50.000 µl

采集方法　　: D:\1260\data\wyz2022\WYZ-ENR22.12.12 2022-12-16 12-12-30\wyz 2020.07.6bayer2BH.M
最后修改　　: 2022/12/16 12:15:58 : 系统
分析方法　　: D:\1260\data\wyz2022\WYZ-ENR22.12.12 2022-12-16 12-12-30\wyz 2020.07.6bayer2BH.M (序列方法)
最后修改　　: 2022/12/17 11:25:27 : 系统
(调用后修改)

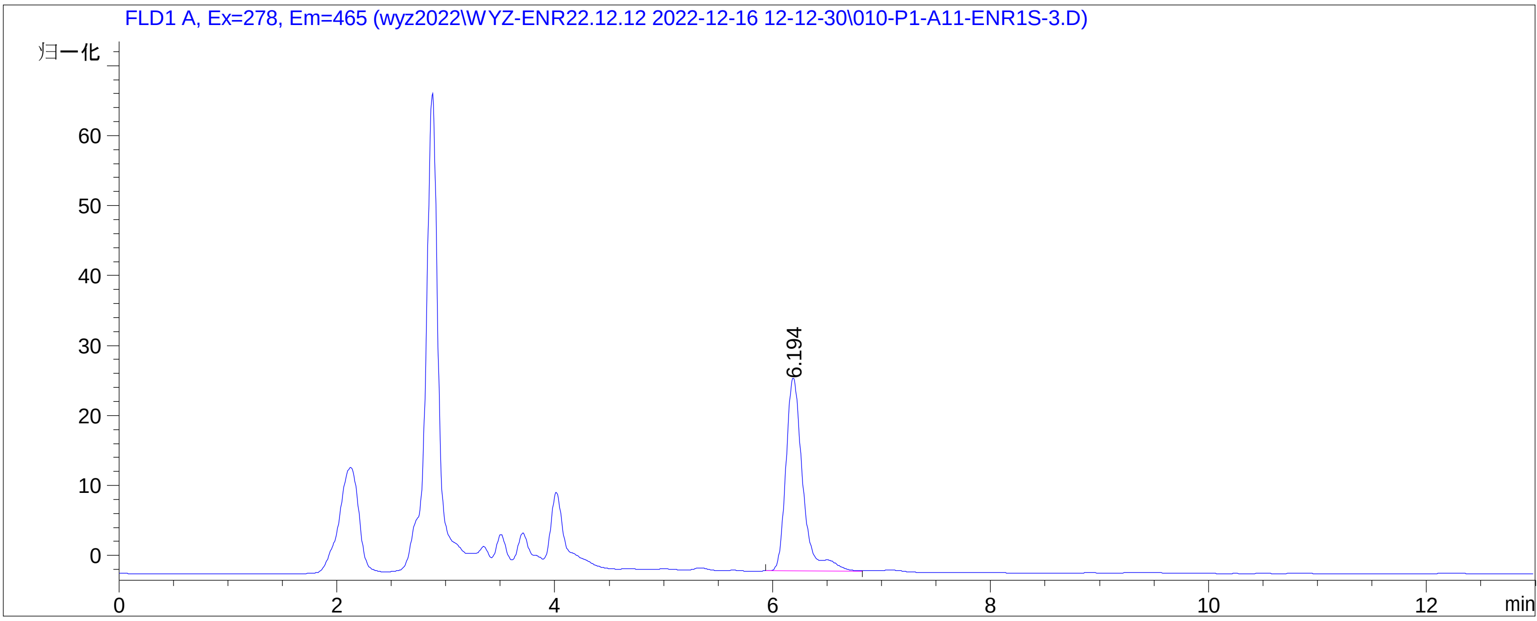

===
面积百分比报告
===

排序　　: 信号
乘积因子　　: 1.0000
稀释因子　　: 1.0000
内标中不使用乘积因子和稀释因子

信号 1: FLD1 A, Ex=278, Em=465

峰 #	保留时间 [min]	类型	峰宽 [min]	峰面积 [LU*s]	峰高 [LU]	峰面积 %
1	6.194	BB	0.1828	269.03738	23.96567	100.0000

总量 :　　269.03738　　23.96567

===
*** 报告结束 ***
