# Supplementary material for: PK-PD integration of enrofloxacin and cefquinome alone and in combination against Klebsiella pneumoniae using an in vitro dynamic model
Source: Front Pharmacol. 2023 Oct 6;14:1226936. doi: 10.3389/fphar.2023.1226936 (PMC10587432; doi:10.3389/fphar.2023.1226936)
Supplement: Supplementary file 2 [file DataSheet3.PDF]

Table 1 P values of the static and dynamic time-kill curves paired with the control

**Static time-kill curve**							
Dose(mg/L)	P value	Dose(mg/L)	P value	Dose(mg/L)	P value	Dose(mg/L)	P value
0.5 MIC ENR	0.013*	0.5 MIC CEQ	0.029*	0.5 MIC ENR+0.25 MIC CEQ	0.025*	0.5 MIC ENR+0.25 MIC CEQ	0.033*
1 MIC ENR	0.025*	1 MIC CEQ	0.012*	0.5 MIC ENR+0.5 MIC CEQ	0.016*	0.5 MIC ENR+0.5 MIC CEQ	0.018*
2 MIC ENR	0.024*	2 MIC CEQ	0.011*	0.5 MIC ENR+1 MIC CEQ	0.015*	0.5 MIC ENR+1 MIC CEQ	0.009**
4 MIC ENR	0.005**	4 MIC CEQ	0.011*	0.5 MIC ENR+2 MIC CEQ	0.015*	0.5 MIC ENR+2 MIC CEQ	0.009**
8 MIC ENR	0.003**	8 MIC CEQ	0.010*	0.5 MIC ENR+4 MIC CEQ	0.012*	0.5 MIC ENR+4 MIC CEQ	0.011*
**Dynamic time-kill curve**							
Dose(mg/L)	P value	Dose(mg/L)	P value	Dose(mg/L)	P value	Dose(mg/L)	P value
0.2mg/L（Total）	0.017*	1.5mg/L(Split-8h)	0.020*	E-1mg/L(Constant)+C-0.75mg/L(Split)	0.001**	E-2mg/L(Constant)+C-0.75mg/L(Split)	0.012*
0.75mg/L（Total）	0.001**	2mg/L(Split-8h)	0.004**	E-1mg/L(Constant)+C-1.25mg/L(Split)	0.002**	E-2mg/L(Constant)+C-1.25mg/L(Split)	0.004**
1.25mg/L（Total）	0.001**	3mg/L(Split-8h)	0.006**	E-1mg/L(Constant)+C-2mg/L(Split)	0.003**	E-2mg/L(Constant)+C-2mg/L(Split)	0.002**
1.5mg/L（Total）	0.000**	4mg/L(Split-8h)	0.003**	E-1mg/L(Constant)+C-4mg/L(Split)	0.003**	E-2mg/L(Constant)+C-4mg/L(Split)	0.004**
2mg/L（Total）	0.000**	2mg/L(Split-12h)	0.005**	E-1mg/L(Constant)+C-4mg/L(Total)	0.002**	E-2mg/L(Constant)+C-4mg/L(Total)	0.003**
1mg/L（Split）	0.001**	4mg/L(Split-12h)	0.004**				
1.5mg/L（Split）	0.000**						
2mg/L（Split）	0.000**						

E stands for Enrofloxacin, C stands for Cefquinome; * indicates significant difference, ** indicates extremely significant difference.
