# Supplementary material for: PK-PD integration of enrofloxacin and cefquinome alone and in combination against Klebsiella pneumoniae using an in vitro dynamic model
Source: Front Pharmacol. 2023 Oct 6;14:1226936. doi: 10.3389/fphar.2023.1226936 (PMC10587432; doi:10.3389/fphar.2023.1226936)
Supplement: Supplementary file 3 [file DataSheet2.ZIP › Protein sequence and Bacterial Strain Identification Report/IGBN22147-Cls2 Bacterial Strain Identification Report..pdf]

IGBN22147 细菌菌种鉴定报告

理论依据：

16S rDNA 是编码原核生物核糖体小亚基 rRNA（16S rRNA）的 DNA 序列，长度约为 1540bp，存在于所有细菌染色体基因组中。16S rDNA 分子大小适中，突变率小，是细菌系统分类学研究中最常用、最有用的“分子钟”。其序列包含可变区和保守区。保守序列区域反应了生物物种间的亲缘关系，而可变序列区域则能体现物种间的差异。16S rDNA 分子的序列特征为不同分类级别的亲缘系统分类奠定了分子生物学基础。通过测定某细菌的 16S rDNA 序列，然后在数据库（如 NCBI）上进行比对，即可获知与该细菌的 16S rDNA 序列同源性最高的已知序列，为确定该菌种的分类地位提供科学依据。

技术路线：

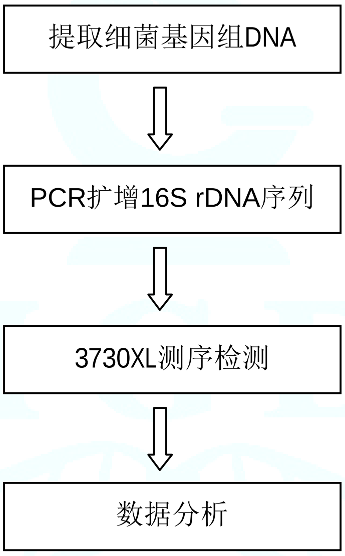

数据报告：

1、测序峰图文件；

2、16S rDNA 序列文件；

3、序列在 NCBI 上的比对数据等。

IGEbio
广州艾基生物技术有限公司

标准产业单元三期四栋 301 室
海珠区，国际生物岛
广州市，广东省
Email: ige_gene@163.com
Telephone: 020-89053723

客户信息：姓　　名 ： 魏彦哲

单位名称 ： 华南农业大学

送检样品形式： 培养皿

送检样品名称： Cls2

检测数据： 测序峰图文件及 16S rDNA 序列文件见附件

拼接序列：

AGCGCCCTCCCGAAGGTTAAGCTACCTACTTCTTTTGCAACCCACTCCCATGGTGTGACGGGCGG
TGTGTACAAGGCCCGGGAACGTATTCACCGTAGCATTCTGATCTACGATTACTAGCGATTCCGACT
TCATGGAGTCGAGTTGCAGACTCCAATCCGGACTACGACATACTTTATGAGGTCCGCTTGCTCTC
GCGAGGTCGCTTCTCTTTGTATATGCCATTGTAGCACGTGTGTAGCCCTGGTCGTAAGGGCCATGA
TGACTTGACGTCATCCCCACCTTCCTCCAGTTTATCACTGGCAGTCTCCTTTGAGTTCCCGGCCTA
ACCGCTGGCAACAAAGGATAAGGGTTGCGCTCGTTGCGGGACTTAACCCAACATTTCACAACAC
GAGCTGACGACAGCCATGCAGCACCTGTCTCACAGTTCCCGAAGGCACCAATCCATCTCTGGAA
AGTTCTGTGGATGTCAAGACCAGGTAAGGTTCTTCGCGTTGCATCGAATTAAACCACATGCTCCA
CCGCTTGTGCGGGCCCCCGTCAATTCATTTGAGTTTTAACCTTGCGGCCGTACTCCCCAGGCGGT
CGATTTAACGCGTTAGCTCCGGAAGCCACGCCTCAAGGGCACAACCTCCAAATCGACATCGTTTA
CGGCGTGGACTACCAGGGTATCTAATCCTGTTTGCTCCCCACGCTTTCGCACCTGAGCGTCAGTC
TTTGTCCAGGGGGCCGCCTTCGCCACCGGTATTCCTCCAGATCTCTACGCATTTCACCGCTACAC
CTGGAATTCTACCCCCCTCTACAAGACTCTAGCCTGCCAGTTTCGAATGCAGTTCCCAGGTTGAG
CCCGGGGATTTCACATCCGACTTGACAGACCGCCTGCGTGCGCTTTACGCCCAGTAATTCCGATT
AACGCTTGCACCCTCCGTATTACCGCGGCTGCTGGCACGGAGTTAGCCGGTGCTTCTTCTGCGG
GTAACGTCAATCGACGAGGTTATTAACCTCACCGCCTTCCTCCCCGCTGAAAGTGCTTTACAACC
CGAAGGCCTTCTTCACACACGCGGCATGGCTGCATCAGGCTTGCGCCCATTGTGCAATATTCCCC
ACTGCTGCCTCCCGTAGGAGTCTGGACCGTGTCTCAGTTCCAGTGTGGCTGGTCATCCTCTCAGA
CCAGCTAGGGATCGTCGCCTAGGTGAGCCGTTACCCCACCTACTAGCTAATCCCATCTGGGCACA
TCTGATGGCATGAGGCCCGAAGGTCCCCCACTTTGGTCTTGCGACATTATGCGGTATTAGCTACC
GTTTCCAGTAGTTATCCCCCTCCATCAGGCAGTTTCCCAGACATTACTCACCCGTCCGCCGCTCGT
CACCCGAGAGCAAGCTCTCTGTGCTACCGCTCGACTTGCA

IGEbio
广州艾基生物技术有限公司

标准产业单元三期四栋 301 室
海珠区，国际生物岛
广州市，广东省
Email: ige_gene@163.com
Telephone: 020-89053723

检测报告：

16S 区 序列在 GenBank 中的比对结果如下：

	Description	Scientific Name	Max Score	Total Score	Query Cover	E value	Per. Ident	Acc. Len	Accession
☑	Klebsiella pneumoniae strain DSM 30104 16S ribosomal RNA, partial sequence	Klebsiella pneumoniae	2586	2586	100%	0.0	99.86%	1530	NR_117686.1
☑	Klebsiella pneumoniae strain JCM1662 16S ribosomal RNA, partial sequence	Klebsiella pneumoniae	2582	2582	100%	0.0	99.79%	1449	NR_112009.1
☑	Klebsiella pneumoniae strain DSM 30104 16S ribosomal RNA, partial sequence	Klebsiella pneumoniae	2580	2580	100%	0.0	99.79%	1530	NR_117684.1
☑	Klebsiella pneumoniae strain ATCC 13883 16S ribosomal RNA, partial sequence	Klebsiella pneumoniae	2579	2579	100%	0.0	99.72%	1452	NR_119278.1
☑	Klebsiella pneumoniae strain NBRC 14940 16S ribosomal RNA, partial sequence	Klebsiella pneumoniae	2577	2577	100%	0.0	99.64%	1465	NR_113702.1

小结：16S 区与 NCBI 数据库比对的结果中，与 *Klebsiella pneumoniae* 的相似性最高，匹配度达到 99.86%。

<u>说明</u>：

再次感谢您使用我们公司的服务，您的实验数据我们将为您保存 1 个月，您提供的样品我们将保存 1 个月，请您拿到样品后仔细阅读此说明，并尽快验证实验结果，如有任何疑问请尽快与我们联系。谢谢您的支持与合作！欢迎您再次使用我们的服务！

实验员：李木兰　　审核员：张海鑫　　日期： 2022-09-22
